# Comparison of acute effects of 3,4-methylenedioxymethamphetamine (MDMA) with and without a supplemental booster dose in healthy participants: a double-blind, randomized, placebo-controlled, crossover study

**DOI:** 10.1038/s41398-026-04148-6

**Published:** 2026-06-04

**Authors:** Mélusine Humbert-Droz, Anna M. Becker, Jan Valenta, Deborah Rudin, Dino Luethi, Ina Vukalovic, Anne Eckert, Matthias E. Liechti, Lorenz Mueller

**Affiliations:** 1 Division of Clinical Pharmacology and Toxicology, Department of Biomedicine and Department of Clinical Research, University Hospital Basel and University of Basel, Basel, Switzerland; 2 Department of Pharmaceutical Sciences, University of Basel, Basel, Switzerland; 3 Psychiatric University Hospital, University of Basel, Basel, Switzerland; 4 Transfaculty Research Platform Molecular and Cognitive Neuroscience, University of Basel, Basel, Switzerland

**Keywords:** Human behaviour, Physiology

## Abstract

3,4-Methylenedioxymethamphetamine (MDMA)-assisted psychotherapy (MDMA-AT) is being investigated as a treatment for several psychiatric disorders, particularly posttraumatic stress disorder. Phase 2 and 3 trials typically administered a first dose of MDMA followed by a second “booster” dose 1.5–2.5 h later, to extend the acute effect duration. However, the risks and benefits of the booster dose have not been systematically investigated. In this double-blind, randomized, placebo-controlled, cross-over study, we compared 120 mg MDMA followed by a 60 mg booster dose or placebo after 2 h, and placebo followed by placebo. The primary outcome was the overall duration of any subjective drug effects, measured by a Visual Analog Scale. Secondary outcomes included additional subjective effects, adverse effects, vital signs, and plasma concentrations of MDMA, oxytocin, and neurophysin I. Twenty-five healthy volunteers were included, and twenty-three (12 male, 11 female) completed all dosing sessions. The booster dose of MDMA prolonged the acute subjective effects of MDMA compared with the single dose (mean ± SD, 5.6 ± 1.8 h *vs*. 4.6 ± 1.2 h, *p* = 0.001), while no differences in subjective or autonomic peak effects were observed. Acute (0–9 h) and subacute (up to 3 days) adverse effects were more common after both MDMA conditions than placebo. These results indicate that acute MDMA effects can be prolonged by using a booster dose, as intended in clinical trials of MDMA-AT. Whether this translates into clinical benefit remains to be investigated.

## Introduction

3,4-Methylenedioxymethamphetamine (MDMA) produces strong acute emotional effects primarily via the release of serotonin, norepinephrine, and oxytocin [1, 2]. MDMA-assisted psychotherapy (MDMA-AT) is currently investigated as a treatment for posttraumatic stress disorder [3, 4], depressive disorder [5], autism spectrum disorder [6], and alcohol use disorder [7]. Clinical Phase 2 and 3 trials typically included two to three dosing sessions with MDMA administered as a split dose: an initial full dose followed by a second “booster” after 1.5–2.5 h [3, 4, 8–14]. An 80 mg dose followed by a 40 mg booster was administered during the first session, increasing to 120 mg plus 60 mg in all subsequent ones, making this the most widely used regimen in MDMA-AT trials.

The booster dose was intended to prolong the acute subjective effects and extend the therapeutic session [8, 11, 14], but its effects have not been systematically investigated. MDMA exhibits marked acute pharmacological tolerance [15], indicated by the observation that acute subjective and autonomic measures return to baseline within 3–5 h while plasma concentrations remain high beyond this time [16, 17]. This has been attributed to transporter-mediated presynaptic monoamine release and neurotransmitter depletion [1, 18]. It is unclear whether raising plasma MDMA levels with a booster dose extends its acute pharmacodynamic effects. However, this acute tolerance is not complete. Two consecutive 100 mg MDMA doses that were separated by 4 h produced two very similar effect-time profiles (the subjective effects returned to baseline before the second dose) while peak plasma concentrations doubled after the second dose [15]. When two 100 mg MDMA doses were separated by 24 h, comparable acute effects were also observed, whereas the second dose produced 1.3-fold higher peak plasma levels [19].

To directly test the effects of the split dose approach, the present study compared the effects and tolerability of a single 120 mg MDMA dose followed by either a 60 mg booster dose or placebo after 2 h, as well as placebo followed by placebo. We hypothesized that the booster would extend the duration of acute subjective effects by approximately one hour compared to MDMA followed by placebo.

## Methods and materials

### Study design

The present Phase 1 study was conducted at the University Hospital Basel. It used a double-blind, placebo-controlled, cross-over design with three dosing sessions: (*i*) a split dose of MDMA (120 mg followed by 60 mg), (*ii*) a single dose of MDMA (120 mg followed by placebo), and (*iii*) placebo followed by placebo. The second dose was administered 2 h after the first. The condition sequence was randomized and counter-balanced, and sessions were separated by a washout period of at least 28 days. The study was conducted in accordance with the Declaration of Helsinki and International Conference on Harmonization Guidelines in Good Clinical Practice and approved by the Ethics Committee of Northwest Switzerland and Swiss Federal Office for Public Health. The study was registered at ClinicalTrials.gov (NCT05809271).

Recruitment occurred via clinicaltrials.gov, advertisement, and word of mouth. Physically and mentally healthy volunteers, between 18 and 65 years of age with a body mass index of 18–29 kg/m^2^, were eligible for participation. Exclusion criteria were pregnancy, breastfeeding, past or current presence of any major psychiatric disorder (assessed by a semi-structured interview based on *Diagnostic and Statistical Manual of Mental Disorders five* (DSM-V) diagnostic criteria), first-degree relative history of psychotic disorders, the use of medications that may interfere with MDMA, chronic or acute physical illness (assessed by physical exam, medical history, and hematological and chemical blood analyses), tobacco smoking (>10 cigarettes/day), alcohol consumption (>15 standard drinks/week), and lifetime MDMA use > 20 times or any illicit drug use within 2 months (except cannabis) prior to inclusion. Participants were asked to refrain from any illicit drug use during the study period. A urine drug test was conducted at screening and once during the study. Full eligibility criteria are provided in the Supplementary Methods and Materials. All participants gave written informed consent and received payment for their participation.

### Study drug

The study drug was produced by Pharmacy Dr. Hysek, Biel, Switzerland, in accordance with Good Manufacturing Practice. Racemic MDMA was formulated as oral capsules that contained 20 mg MDMA hydrochloride. The exact content, determined by high-performance liquid chromatography, was 20.74 ± 0.47 mg (mean ± SD, *n* = 10). The placebo consisted of matching capsules that contained mannitol. Randomization, packaging, labeling, and quality control were performed by the Good Manufacturing Practice producer.

### Study procedures

After providing written informed consent, the eligibility of participants was assessed at a screening visit by trained study personnel and a study physician. Included participants attended three 10-h dosing sessions and an end-of-study visit. Dosing sessions started at 8 AM and were conducted in a quiet hospital room that was equipped with a bed. Upon arrival, baseline and safety measurements were taken (i.e., adverse events since the last visit, vital parameters, and urine drug and/or pregnancy tests), and participants were served a standardized breakfast. The first dose was administered around 9 AM, with the second dose exactly 2 h later. Outcome measures (autonomic effects, questionnaires, and blood samples) were repeatedly assessed over 9 h. Supplementary Table S1 summarizes the time course of outcome assessments during the sessions. Participants remained under continuous supervision during the acute effect phase. They were allowed to listen to music of their choice, had access to a balcony, and could go on a short walk accompanied by their supervisor. After a 10-h stay, the participants were discharged home. Additional questionnaires were completed at home 3 and 7 days after dosing.

### Blinding

Participants guessed their treatment assignment at the end of each dosing session and after study completion. They knew about the administered conditions/doses but not the sequence. All participants and study personnel remained blinded until the end-of-study visit.

### Subjective effects

Single-item Visual Analog Scales (VASs), presented as 100-mm horizontal lines (0–100%) and marked from “not at all” on the left to “extremely” on the right, were repeatedly administered to assess drug-induced acute subjective effects over time. They included “any drug effect,” “good drug effect,” “bad drug effect,” “drug-liking,” “feeling stimulated,” “drug-high,” and “anxiety.” Further items were presented as bidirectional scales (−50% to +50%), with 0 defined as baseline before drug administration. These items included “emotional,” “talkative,” “happy,” “open,” “trust,” “feeling close to others,” “I want to be alone,” and “I want to be with others.” The items “any drug effect,” “good drug effect,” and “bad drug effect” were queried every 15 min throughout the session, and the rest were administered 0, 0.5, 1, 1.5, 2, 2.5, 3, 3.5, 4, 4.5, 5, 5.5, 6, 7, 8, and 9 h after drug administration. Maximal ratings (E_max_) and areas under the effect-time curve (AUECs) were calculated for each VAS item. Effect duration was defined as the cumulative time the item “any drug effect” remained over a threshold of 10%.

Changes in mood were also assessed with the Adjective Mood Rating Scale (AMRS) before and 2, 4, 6, and 9 h after dosing [20]. The VASs and AMRS are described in detail in the Supplementary Methods and Materials.

### Adverse effects

Acute and subacute adverse effects were assessed using the revised List of Complaints (LC) [21], which consists of 40 items rated on a four-point intensity scale from 0 (not at all) to 3 (strong). Additional 10 items of frequently observed adverse effects after MDMA administration were added to this list, resulting in a total of 50 items. The List of Complaints was administered at baseline (0 h), at the end of the dosing session (0–9 h), and at 3 and 7 days post-session, each covering the period since the last assessment. Additional adverse effects that were not, or not adequately, captured by the LC, occurred outside these intervals, or required medical treatment were recorded on separate standardized forms.

Mental well-being and subjective sleep quality were assessed with the General Health Questionnaire-12 (GHQ-12) [22], the Warwick-Edinburgh Mental Well-Being Scale (WEMWBS) [23], and the Insomnia Severity Index (ISI) [24] administered at baseline and 3 and 7 days after drug administration, referring to the previous 3 and 4 days since the last assessment, respectively. Higher GHQ-12 scores (range, 0–36) indicated greater distress. Higher WEMWBS scores (range, 14–70) indicated greater well-being. Higher ISI scores (range, 0–28) indicated poorer sleep. Total scores for each condition and timepoint were calculated and compared between conditions. Details are provided in the Supplementary Methods and Materials.

### Blood sampling and autonomic effects

Plasma concentrations of MDMA and its metabolites 3,4-methylenedioxyamphetamine (MDA) and 4-hydroxy-3-methoxymethamphetamine (HMMA) were measured every 30 min for 6 h after dosing and hourly thereafter using a validated method [25]. Blood pressure, heart rate, and tympanic temperature were recorded at the same timepoints. Plasma concentrations of oxytocin and its carrier protein neurophysin I were measured before and 2, 4, and 6 h after dosing. Blood cell counts and liver/kidney function were assessed at screening and the end-of-study visit. Further details on oxytocin and neurophysin I analyses and routine blood samples are provided in the Supplementary Methods and Materials.

### Pharmacokinetic analyses

The pharmacokinetic parameters of MDMA, MDA, and HMMA were calculated using non-compartmental analyses in Phoenix WinNonlin 8.4 (Certara, Princeton, NJ, USA). The exact sampling times and linear-up/log down method were used. Details are provided in the Supplementary Methods and Materials.

### Statistical analyses and sample size estimation

The primary endpoint of the study was the difference in effect duration of the VAS item “any drug effect” between conditions. A sample size of 20 participants was calculated to provide 80% power to detect a within-subject difference of moderate effect size (Cohen’s d ≈ 0.66), assuming a two-sided significance level (α = 0.05). Statistical analyses were performed using jamovi software (the jamovi project, version 2.5, 2024) and KNIME 5.2.5 software (KNIME AG, Zurich, Switzerland). Differences between conditions were assessed using repeated-measures analysis of variance (ANOVA) with condition as within-subject factor, followed by Tukey’s *post hoc* tests for multiple comparisons. All tests were two-sided, and the level of significance was *p* < 0.05.

To further characterize adverse effects, post hoc exploratory analyses stratified by sex, body weight (<65 and ≥65 kg), and age (<30 and ≥30 years) were conducted using descriptive statistics.

## Results

### Participants and blinding

The study was conducted between November 17, 2023, and March 25, 2025. Twenty-five participants were included and completed at least one session. Two participants dropped out after the first session, resulting in a final sample size of 23 (12 male, 11 female). Baseline characteristics are described in Supplementary Table S2. The mean ± SD age was 35 ± 13 years. Body weight was 67 ± 12 kg. Ten participants (43%) had prior MDMA experience (5.3 ± 3.8 times).

All participants correctly identified the placebo condition. Overall, 35 of the 46 (76%) MDMA conditions (single dose and booster dose) were correctly identified directly after the dosing session, and 40 of the 46 MDMA conditions (87%) were correctly identified at the end of the study (Supplementary Table S3).

### Acute subjective effects

**Fig. 1:**
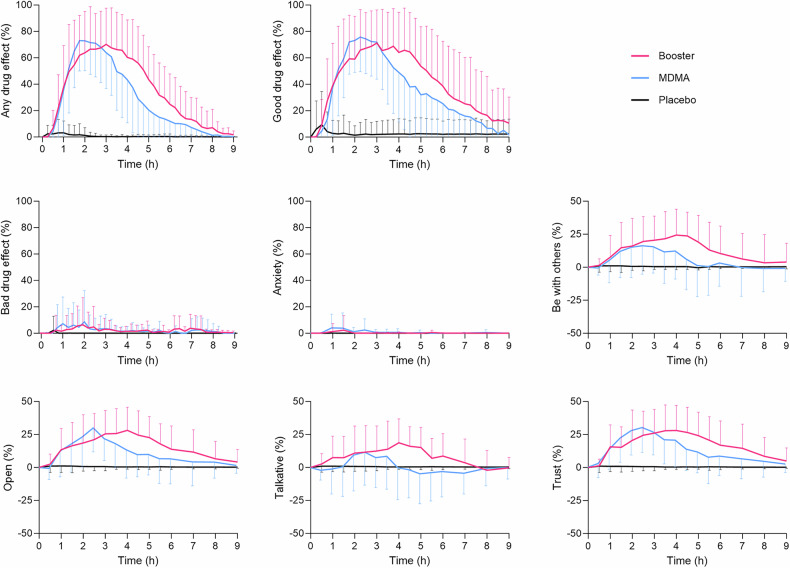
Results from single-item Visual Analog Scales over time. The data are expressed as mean scores (%) with standard deviations as error bars. *n* = 23.

Acute subjective effects are presented in Table 1, Fig. 1, Supplementary Table S4, and Supplementary Figure S1. The primary endpoint measure, the duration of “any drug effect,” was significantly longer after the booster dose (mean ± SD, 5.6 ± 1.8 h) compared with the MDMA single dose (4.6 ± 1.2 h; *p* = 0.001). Peak VAS intensity did not differ between the two MDMA conditions (Table 1). Other subjective effects were similarly prolonged after the booster dose, reflected by higher AUEC values. Consistent with these findings, “well-being” scores on the AMRS increased at the 4 and 6 h timepoints after the booster dose compared with the MDMA single dose (Supplementary Table S5 and Supplementary Figure S3). Acute pharmacological tolerance could be observed for both MDMA conditions and is illustrated in a hysteresis plot (Supplementary Figure S2).

**Table 1:** Acute subjective effects on single-item Visual Analog Scales.

Condition		Booster			MDMA			Placebo			F_2, 44_	*p*	Booster - MDMA	Booster - Placebo	MDMA - Placebo
		mean ± SD			mean ± SD			mean ± SD							
**Unidirectional scales (0–100%)**															
Any drug effect	E_max_	83	±	21	85	±	20	4.8	±	11	241	<0.001	NS	***	***
	AUEC	318	±	127	255	±	130	6.1	±	23	88.5	<0.001	*	***	***
	Duration (h)	5.6	±	1.8	4.6	±	1.5	0.2	±	0.5	173	<0.001	**	***	***
Good drug effect	E_max_	86	±	29	87	±	19	10	±	26	151	<0.001	NS	***	***
	AUEC	373	±	186	309	±	191	23	±	95	59.5	<0.001	NS	***	***
Bad drug effect	E_max_	18	±	24	17	±	27	2.3	±	11	5.41	<0.01	NS	**	*
	AUEC	19	±	32	20	±	43	0.6	±	2.7	4.35	<0.05	NS	*	NS
Anxiety	E_max_	4.2	±	12	7.0	±	14	0.1	±	0.3	4	<0.05	NS	NS	NS
	AUEC	2.6	±	6.7	7.8	±	17	0.0	±	0.1	4.61	<0.05	NS	NS	NS
**Bidirectional scales (−50% to** + **50%)**															
Be with others	E_max_	29	±	19.2	24	±	21	1.2	±	5.0	25.5	<0.001	NS	***	***
	E_min_	−8.3	±	15.6	−9.7	±	17	−0.3	±	1.7	5.55	<0.01	NS	NS	*
	AUEC	110	±	120	47	±	126	4.1	±	19	11.1	<0.001	**	***	NS
Open	E_max_	34	±	16	35	±	15	1.1	±	5.0	79.3	<0.001	NS	***	***
	E_min_	−3.2	±	7.9	−7.6	±	14	0.0	±	0.0	5.57	<0.01	NS	NS	*
	AUEC	139	±	95	94	±	97	4.6	±	22	27.7	<0.001	*	***	***
Talkative	E_max_	28	±	17	23	±	19	1.0	±	4.6	25.9	<0.001	NS	***	***
	E_min_	−13	±	15	−17	±	18	0.0	±	0.0	13.9	<0.001	NS	**	***
	AUEC	69	±	71	3.5	±	110	4.7	±	22	6.82	<0.01	*	***	NS
Trust	E_max_	34	±	18	36	±	15	1.1	±	4.8	75.3	<0.001	NS	***	***
	E_min_	−1.5	±	6.7	−2.7	±	10	0.0	±	0.0	0.772	0.468	NS	NS	NS
	AUEC	153	±	109	118	±	98	4.5	±	21	35.3	<0.001	*	***	***

A threshold of 10% was set to calculate effect duration. The MDMA condition consisted of an MDMA single dose (120 mg), the Booster condition was a 180 mg split dose (120 mg followed by 60 mg after 2 h). AUECs for bidirectional VASs were calculated by subtracting the AUEC below baseline from the AUEC above baseline. Repeated Measures Analyses of Variance (ANOVAs) followed by tukey post hoc tests were used.

*SD* standard deviation, *AUEC* area under the effect-time curve, *E*_*max*_ maximal effects, *E*_*min*_ minimal effects.

*p < 0.05, **p < 0.01, ***p < 0.001; NS, not significant, *n* = 23.

### Acute and subacute adverse effects

Somatic and psychological adverse effects, assessed by the List of Complaints, GHQ-12, WEMWBS, and ISI, are presented in Table 2 and Supplementary Figure S4. Intensity-graded List of Complaints scores indicated increases in acute (0–9 h) and subacute (days 0–3) complaints after both MDMA conditions *vs*. placebo, with no significant differences between booster and single dose. Differences from baseline List of Complaints scores were nominally higher after the booster compared with the single dose of MDMA, but this difference did not reach statistical significance. Intensity-graded and overall frequencies of each symptom on the List of Complaints are reported in Supplementary Tables S6 and S7, respectively. The most frequent acute adverse effects were tiredness, lack of appetite, bruxism, and headache. Emesis occurred in three participants (13%) during the booster condition and in two participants (9%) during the single-dose condition. Somatic complaints, such as a lack of appetite, bruxism, and perspiration, diminished by days 3 and 7, whereas psychological symptoms, such as rumination, fatigue, inner tension, and feeling restless, persisted to days 3 and 7 (Supplementary Tables S6 and S7). Total scores of the List of Complaints, stratified by sex, weight, and age, are listed in Supplementary Table S8. Acute and subacute List of Complaints scores were nominally higher in women and in participants with low body weight (<65 kg), particularly after the booster dose.

**Table 2:** Acute and subacute adverse effects.

Condition	Booster	MDMA	Placebo	*F*_2, 44_	*p*	Booster - MDMA	Booster - Placebo	MDMA - Placebo
	mean ± SD	mean ± SD	mean ± SD					
**List of complaints**								
Baseline	5.2 ± 6.1	8.4 ± 14	5.6 ± 6.4	1.12	NS	NS	NS	NS
Acute (0–9 h)	22 ± 15	20 ± 13	4.4 ± 5.3	24.6	<0.001	NS	***	***
Subacute (Days 0–3)	16 ± 16	17 ± 16	7.7 ± 13	9.99	<0.001	NS	***	**
Subacute (Days 4–7)	6.9 ± 9.8	9.6 ± 13	6.9 ± 11	1.87	NS	NS	NS	NS
**General Health Questionnaire-12**								
Baseline	9.7 ± 3.3	10 ± 3.6	8.1 ± 1.9	3.98	<0.05	NS	NS	*
Subacute (Days 0–3)	9.4 ± 4.6	11 ± 5.1	10 ± 4.3	1.3	NS	NS	NS	NS
Subacute (Days 4–7)	8.2 ± 2.6	9.4 ± 3.5	9.9 ± 3.5	2.55	NS	NS	NS	NS
**Warwick-Edinburgh Mental Well-being Scale**								
Baseline	55 ± 5.6	54 ± 3.9	55 ± 5.1	0.659	NS	NS	NS	NS
Subacute (Days 0–3)	53 ± 6.8	49 ± 7.9	54 ± 7.6	3.7	<0.05	NS	NS	NS
Subacute (Days 4–7)	54 ± 6.0	54 ± 5.0	54 ± 6.3	0.202	NS	NS	NS	NS
**Insomnia Severiy Index**								
Baseline	5.0 ± 3.5	5.9 ± 4.8	4.1 ± 4.0	2.27	NS	NS	NS	NS
Subacute (Days 0–3)	4.5 ± 3.6	5.6 ± 4.6	4.0 ± 3.9	1.5	NS	NS	NS	NS
Subacute (Days 4–7)	2.7 ± 2.2	4.4 ± 3.7	4.6 ± 3.9	5.76	<0.01	*	**	NS

The MDMA condition consisted of a MDMA single dose (120 mg), the Booster condition was a 180 mg split dose (120 mg followed by 60 mg after 2 h). Repeated Measures Analyses of Variance (ANOVAs), followed by Tukey post hoc tests were used. Results are shown as mean ± SD total scores.

*MDMA* 3,4-methylenedioxymethamphetamine, *SD* standard deviation.

*p < 0.05; **p < 0.01; ***p < 0.001; NS, not significant, *n* = 23.

GHQ-12 and WEMWBS scores showed no differences between conditions, and ISI scores indicated slightly better sleep 4–7 days after the booster.

There was one serious adverse event during the study. One participant developed acute angle-closure glaucoma during the single-dose MDMA session, requiring emergency referral to the ophthalmologic clinic with an overnight stay. The participant showed complete recovery after adequate treatment.

Two participants dropped out after completing the first session. Both were women, and both had received the booster condition, which was also their first ever MDMA experience. Both participants experienced intense headaches and nausea during the session, followed by mental instability in the following weeks. The first participant reported symptoms of anxiety, confusion, and impaired concentration, resulting in a reduced ability to work. Symptoms improved gradually over several months. The second participant reported anxiety, restlessness, and tension the day after the treatment session, which worsened for one week and then stabilized for another week before slowly improving. She experienced fear of being alone, requiring family support. At the end-of-study visit, almost 2 months after the study session, she reported having returned to normal. Both participants were provided with psychological support and were followed up on a regular basis. List of Complaints scores of these two dropout participants are listed in Supplementary Table S9, as they are excluded from within-subject comparisons. Two male participants (9%) reported flashback-like experiences, described as a pleasant reoccurrence of mild effects similar to their MDMA session, with one participant also experiencing palpitations. These events began the day after the study session, lasted several minutes, and occurred at irregular intervals that gradually became longer before resolving.

### Endocrine effects

**Fig. 2:**
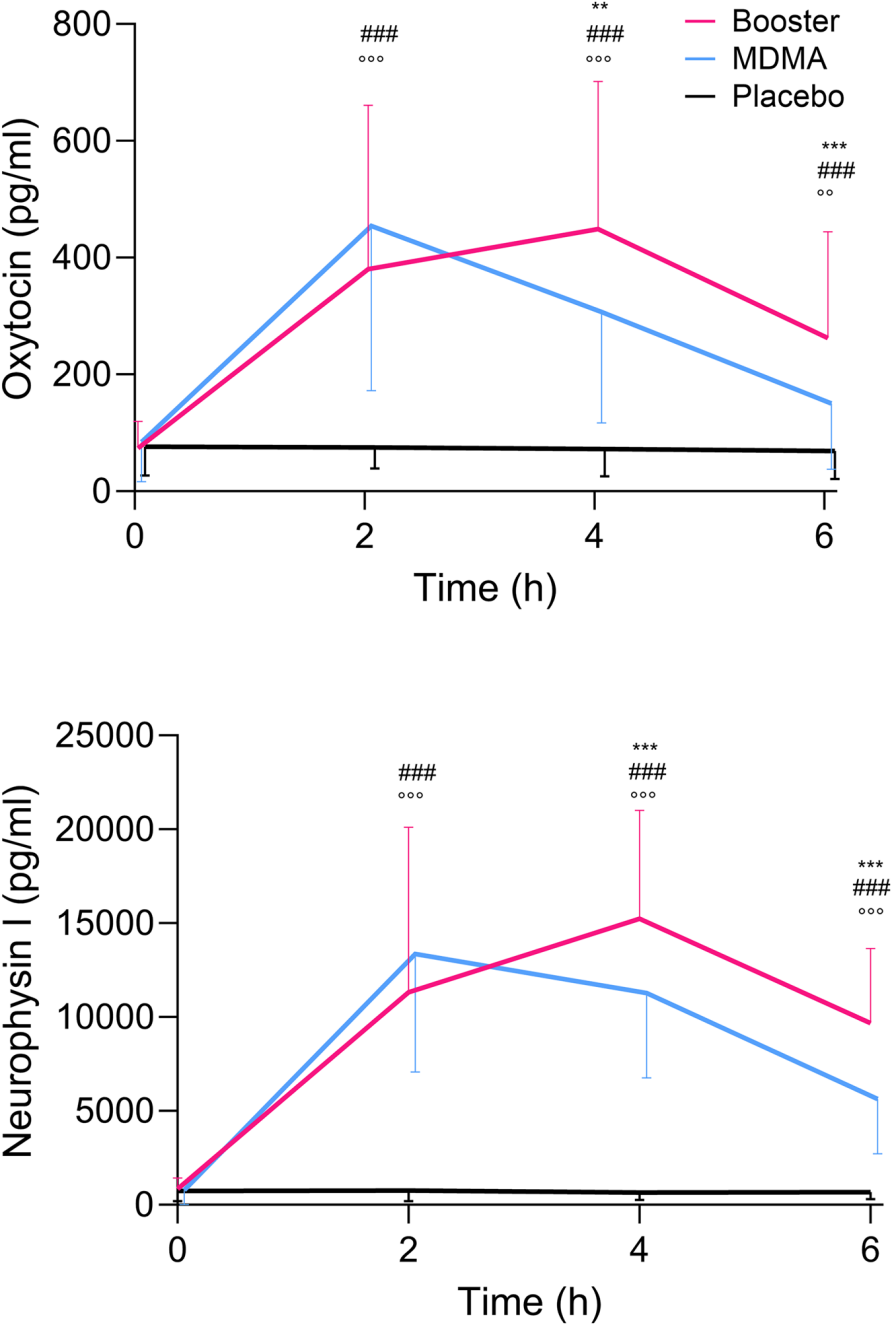
Oxytocin and neurophysin I concentrations (mean with standard deviation as error bars) at different time points. *P < 0.05, **P < 0.01, ***P < 0.001 Booster compared to MDMA, #*P* < 0.05, ##*P* < 0.01, ###*P* < 0.001 Booster compared to *P*lacebo, °*P* < 0.05, °°*P* < 0.01, °°°*P* < 0.001 MDMA compared to Placebo. *n* = 23.

Plasma oxytocin and neurophysin I concentrations are shown in Fig. 2, Supplementary Figure S5, and Supplementary Table S10. Both MDMA conditions significantly increased circulating oxytocin and neurophysin I compared with placebo. The booster dose significantly increased both oxytocin and neurophysin I concentrations at 4 and 6 h compared with single-dose MDMA. Oxytocin and neurophysin I concentrations positively correlated with each other (Pearson correlation, *r* = 0.86, *p* < 0.0001). Linear regression analysis indicated that for every 1000 pg/ml increase in neurophysin I, oxytocin concentrations increased by approximately 30 pg/ml (Supplementary Figure S5).

### Autonomic effects

Blood pressure, heart rate, rate-pressure product, and body temperature increased under both MDMA conditions *vs*. placebo, with a longer duration but not a higher maximal effect after the booster (Supplementary Table S11 and Supplementary Figure S6). No incidences of systolic blood pressure exceeding 180 mmHg or hyperpyrexia ( > 40 °C) were observed in any participant.

### Effects on kidney and liver function and changes in blood cell counts

No abnormalities in routine laboratory measures were observed between the screening and end-of-study visits (Supplementary Table S12).

### Pharmacokinetics

Pharmacokinetic parameters of MDMA and its metabolites are summarized in Table 3, and mean and individual plasma concentrations over time are presented in Supplementary Figure S7. The observation time was not long enough to comprehensively describe MDMA elimination pharmacokinetics (see Supplementary Results for a detailed description). MDMA showed dose-proportional increases in C_max_ and AUC_0-9_ values. The elimination half-life of MDMA was 7.2 h in both conditions.

**Table 3:** Pharmacokinetic parameters of MDMA, MDA, and HMMA based on non-compartmental analyses.

Condition	Booster			MDMA			Ratio	
	*n*	Geometric mean (95% CI)	Range	*n*	Geometric mean (95% CI)	Range	*n*	geometric mean ratio (90% CI)
**MDMA**								
C_max_ (ng/ml)	25	387 (351–426)	266–611	23	253 (228–280)	161–408	23	1.50 (1.43–1.57)
*t*_max_ (h)	25	4 (3.7–4.4)	3.0–6.0	23	2.8 (2.5–3.1)	1.6–5.0		
AUC_0-9_ (ng·h/ml)	25	2339 (2151–2543)	1658–3313	23	1564 (1416–1726)	1061–2278	23	1.47 (1.40–1.54)
*t*_1/2_ (h)	16	7.2 (6.2–8.3)	5.1–14	15	7.2 (6.0–8.6)	3.7–11		
CL/F (L/h)	16	34 (30–39)	23–57	15	39 (32–47)	24–76		
V_z_/F (L)	16	353 (317–393)	257–493	15	402 (355–456)	258–568		
**MDA**								
C_max_ (ng/ml)	25	20 (18–22)	9.6–27	23	13 (12–15)	7.7–21	23	1.52 (1.44–1.60)
AUC_0-9_ (ng·h/ml)	25	112 (100–126)	57–172	23	81 (72–92)	50–143	23	1.36 (1.28–1.45)
**HMMA**								
C_max_ (ng/ml)	25	92 (73–116)	30–244	23	85 (64–113)	26–261	23	1.09 (1.02–1.17)
*t*_max_ (h)	25	4 (3.4–4.7)	1.5–8.1	23	3.2 (2.6–3.8)	1.0–7.0		
AUC_0-9_ (ng·h/ml)	25	618 (489–780)	191–1526	23	561 (426–739)	175–1604	23	1.11 (1.05–1.18)
*t*_1/2_ (h)	11	7.4 (6.5–8.3)	5.8–9.8	14	6.9 (6.0–8.0)	4.9–11		

Dropouts were retained in the analysis resulting in *n* = 25 for the Booster condition and *n* = 23 for the MDMA condition. The MDMA condition consisted of a MDMA single dose (120 mg), the Booster condition was a 180 mg split dose (120 mg followed by 60 mg after 2 h).

*MDMA*, 3,4-methylenedioxymethamphetamine; *MDA*, 3,4-methylenedioxyamphetamine; *HMMA*, 4-Hydroxy-3-methoxymethamphetamine; *CI*, confidence interval; *AUC*_*0-9*_, area under the plasma concentration-time curve from time 0 to 9 h; *CL/F* apparent total clearance; *C*_*max*_, maximum observed plasma concentration; *t*_1/2_, plasma half-life; *t*_max_, time to reach C_max_; *V*_*z*_*/F*, apparent volume of distribution; *n*, number of included profiles.

## Discussion

The present study compared an MDMA split dose (120 mg + 60 mg booster after 2 h) with a single 120 mg dose followed by placebo and placebo followed by placebo. Although similar booster doses have been used in most published Phase 2 and 3 trials [3, 4, 6, 8–14, 26, 27], their pharmacodynamic effects have not been previously evaluated systematically. The main finding of the present study was that the booster dose prolonged the subjective effect duration by an average of 1 h compared with the single dose, from 4.6 to 5.6 h, without increasing maximal effects. This prolongation was also reflected by significantly higher AUEC values for several VAS items, including “drug high,” “drug liking,” “be with others,” “feeling close to others,” “open,” “talkative,” and “trust,” in the MDMA booster condition compared with the single dose. The MDMA booster dose also prolonged elevations of blood pressure, heart rate, and oxytocin, with no difference in maximal values, aligning with the extended duration of subjective effects.

The peak plasma concentration and total exposure (AUC_0-9_) of MDMA increased dose-proportionality after the booster dose, while the elimination half-life of MDMA remained unchanged (7.2 h). The time to reach maximal MDMA plasma concentrations (C_max_) was 3 and 4 h in the single-dose and booster dose conditions, respectively. A difference in T_max_ of 2 h could have been expected, given administration of the booster 2 h after the first dose. Participants were served breakfast before the first dose, which might have slowed absorption of the first dose of MDMA, whereas the booster dose was possibly absorbed more rapidly.

The lack of differences in maximal subjective and autonomic responses despite higher plasma concentrations after the booster dose is consistent with acute pharmacodynamic tolerance, a known phenomenon after MDMA administration. Subjective and physiological effects subsided within the 9 h observation period while MDMA concentrations remained high (mean plasma concentration at discharge after the booster dose exceeded mean peak plasma levels after the single dose). The depletion of intracellular transmitter pools has been proposed to contribute to acute tolerance [1, 18]. Prior work with repeated MDMA dosing showed partial acute tolerance. Two 100 mg doses that were separated by 4 h produced two very similar subsequent effect-time profiles (subjective effects had returned to baseline before the second dose), while peak MDMA concentrations doubled [15]. In the present study, the booster dose prolonged acute effects of MDMA by 1 h. Maximal MDMA plasma concentrations were also reached with a delay of 1 h after the booster compared with the single dose. Altogether, the findings of the present study and prior studies [15] indicate that the acute response to MDMA can be prolonged despite acute tolerance, and redosing might prolong acute responses even further by increasing the re-dosing interval.

A range of potential toxic effects related to MDMA has been described. These include increased off-target dopaminergic activity leading to abuse liability and neurotoxicity [28, 29], 5HT-2B receptor activation potentially promoting endocardial fibrosis [30, 31], hepatic toxicity [32], and hyperthermia [33]. These effects would be expected to increase dose-dependently; however, in the present study, autonomic effects were moderate and lower than anticipated when considering the relatively high total dose of 180 mg [34, 35]. The booster did not significantly increase acute or subacute adverse effects overall, but List of Complaints scores were nominally higher after booster dosing. Most adverse effects were mild and transient, but two participants (8%) discontinued the study after receiving the booster as their first condition and experiencing strong acute symptoms (headache, nausea) followed by persistent psychological symptoms. Unclear is whether the single dose might have produced less adverse effects in these participants. Importantly, both participants had high List of Complaints scores that were not included in the within-subject comparison and are reported separately (Supplementary Table S9). In exploratory analyses, LC scores appeared higher in women and participants <65 kg, particularly after the booster dose and during the acute (0–9 h) phase. In addition, vomiting occurred exclusively in women, all of whom were in a lower weight range (57–63 kg). In our sample, sex and body weight could not be disentangled, due to their strong correlation. These observations warrant further investigation, as lower doses, omission of the booster, or body-weight-adjusted dosing might be safer in certain subgroups. Subacute adverse effects of MDMA, including lower mood states, a lack of energy, and irritability, have been described mostly among recreational Ecstasy users, referred to as “blue Monday” or “mid-week blues” [36–39]. In the present controlled study, we observed higher List of Complaints scores 1–3 days post-dose after both MDMA conditions, consistent with mild subacute effects. However, no lasting change in mental well-being was detected on the GHQ-12 or WEMWBS up to 7 days post-dose.

One serious adverse event occurred during the study, a case of acute angle-closure glaucoma following the MDMA single dose. The participant was subsequently diagnosed with narrow angles, a known risk factor for the development of acute glaucoma in response to mydriatics. The participant showed complete recovery after adequate treatment. Notably, the participant had previously completed both the MDMA booster and placebo sessions without any complications. Glaucoma has previously been described in recreational users of Ecstasy [40, 41] and may be a rare but relevant complication.

Trials on MDMA-AT used the booster dose to extend the therapeutic session. The present study was the first to explicitly investigate the effect of such a booster dose on the acute response to MDMA. Our findings highlight that booster dosing prolongs the acute response to MDMA, including pro-social and affective effects (e.g., openness, trust, and talkativeness) and oxytocin release, which could be advantageous in psychotherapy. However, the added therapeutic value of a booster dose over a single dose remains unproven. Although no acute serious safety concerns arose with administration of the MDMA booster dose in the present study, the total dose of 180 mg may be too high for subgroups of patients. Clinical Phase 2 and 3 trials often already address this by administering a lower MDMA dose during the first session (80 mg + 40 mg booster), with an optional escalation to 120 mg + 60 mg for later sessions [3, 4]. This dosing approach resulted in the full 120 mg + 60 mg dose being used in >95% of patients in recent MDMA-AT trials [3, 4]. The regimen that was tested in the present study thus reflects the regimen that is most commonly used in clinical trials. However, whether this approach is optimal in terms of clinical outcomes remains to be determined. Future research could directly compare alternative dosing strategies, such as a single-dose regimen or weight-adjusted dosing in patient populations. Study limitations include our mostly young and healthy sample, which lacks generalizability, particularly to patient populations with arterial hypertension or other comorbidities, who may exhibit stronger autonomic reactions. Adverse effects were not assessed daily but rather over time intervals of different lengths, which may hamper direct comparison and may be prone to recall bias. Sex and body weight effects could not be disentangled given the correlation of women and body weights <65 kg.

Strengths of the study include the randomized, placebo-controlled, powerful within-subject design, relatively large sample, long washout periods, and the use of a dosing schedule that closely aligned with therapeutic trials. Both acute and subacute effects were comprehensively assessed alongside pharmacokinetics and endocrine markers.

## Conclusion

A 60 mg booster dose of MDMA 2 h after 120 mg MDMA extended subjective, autonomic, and endocrine effects by approximately 1 h without increasing maximal responses. The regimen was overall well tolerated. Whether booster dosing provides meaningful therapeutic benefit in MDMA assisted therapy requires further evaluation in patient populations.

## Supplementary information

Supplement MDMA-Booster

## Data Availability

The datasets in this article are not readily available. Requests to access the datasets should be directed to ML (matthias.liechti@usb.ch).

## References

[1] Hysek CM, Simmler LD, Nicola V, Vischer N, Donzelli M, Krähenbühl S, et al. Duloxetine inhibits effects of MDMA (“ecstasy”) in vitro and in humans in a randomized placebo-controlled laboratory study. PLoS One. 2012;7:e36476. 22574166 10.1371/journal.pone.0036476 PMC3344887

[2] Atila C, Holze F, Murugesu R, Rommers N, Hutter N, Varghese N, et al. Oxytocin in response to MDMA provocation test in patients with arginine vasopressin deficiency (central diabetes insipidus): a single-centre, case-control study with nested, randomised, double-blind, placebo-controlled crossover trial. Lancet Diabetes Endocrinol. 2023;11:454–64. 37192642 10.1016/S2213-8587(23)00120-1

[3] Mitchell, Ot’alora GM JM, van der Kolk B, Shannon S, Bogenschutz M, Gelfand Y, et al. MDMA-assisted therapy for moderate to severe PTSD: a randomized, placebo-controlled phase 3 trial. Nat Med. 2023;29:2473–80. 37709999 10.1038/s41591-023-02565-4 PMC10579091

[4] Mitchell JM, Bogenschutz M, Lilienstein A, Harrison C, Kleiman S, Parker-Guilbert K, et al. MDMA-assisted therapy for severe PTSD: a randomized, double-blind, placebo-controlled phase 3 study. Nat Med. 2021;27:1025–33. 33972795 10.1038/s41591-021-01336-3 PMC8205851

[5] Kvam TM, Goksoyr IW, Rog J, van de Vooren IJ, Stewart LH, Autran I, et al. MDMA-assisted therapy as a treatment for major depressive disorder: proof of principle study. Br J Psychiatry. 2025;227:1–7. 10.1192/bjp.2025.10320 PMC12550655 40641116

[6] Danforth AL, Grob CS, Struble C, Feduccia AA, Walker N, Jerome L, et al. Reduction in social anxiety after MDMA-assisted psychotherapy with autistic adults: a randomized, double-blind, placebo-controlled pilot study. Psychopharmacology (Berl). 2018;235:3137–48. 30196397 10.1007/s00213-018-5010-9 PMC6208958

[7] Thurgur H, Sessa B, Higbed L, O’Brien S, Durant C, Wilson S, et al. MDMA-assisted psychotherapy for AUD: Bayesian analysis of WHO drinking risk level and exploratory analysis of drinking behavior and psychosocial functioning at 3 months follow-up. Alcohol Alcohol. 2025;60:agaf031. 40492610 10.1093/alcalc/agaf031 PMC12148347

[8] Mithoefer MC, Wagner MT, Mithoefer AT, Jerome I, Doblin R. The safety and efficacy of ±3,4-methylenedioxymethamphetamine-assisted psychotherapy in subjects with chronic, treatment-resistant posttraumatic stress disorder: the first randomized controlled pilot study. J Psychopharmacol. 2010;25:439–52. 20643699 10.1177/0269881110378371 PMC3122379

[9] Mithoefer MC, Mithoefer AT, Feduccia AA, Jerome L, Wagner M, Wymer J, et al. 3,4-Methylenedioxymethamphetamine (MDMA)-assisted psychotherapy for post-traumatic stress disorder in military veterans, firefighters, and police officers: a randomised, double-blind, dose-response, phase 2 clinical trial. Lancet Psychiatry. 2018;5:486–97. 29728331 10.1016/S2215-0366(18)30135-4

[10] Ot’alora GM, Grigsby J, Poulter B, Van Derveer JW 3rd, Giron SG, et al. 3,4-Methylenedioxymethamphetamine-assisted psychotherapy for treatment of chronic posttraumatic stress disorder: a randomized phase 2 controlled trial. J Psychopharmacol. 2018;32:1295–307. 30371148 10.1177/0269881118806297 PMC6247454

[11] Oehen P, Traber R, Widmer V, Schnyder U. A randomized, controlled pilot study of MDMA (±3,4-methylenedioxymethamphetamine)-assisted psychotherapy for treatment of resistant, chronic post-traumatic stress disorder (PTSD). J Psychopharmacol. 2013;27:40–52. 23118021 10.1177/0269881112464827

[12] Christie D, Yazar-Klosinski B, Nosova E, Kryskow P, Siu W, Lessor D, et al. MDMA-assisted therapy is associated with a reduction in chronic pain among people with post-traumatic stress disorder. Front Psychiatry. 2022;13:939302. 36405923 10.3389/fpsyt.2022.939302 PMC9669702

[13] Nicholas CR, Wang JB, Coker A, Mitchell JM, Klaire SS, Yazar-Klosinski B, et al. The effects of MDMA-assisted therapy on alcohol and substance use in a phase 3 trial for treatment of severe PTSD. Drug Alcohol Depend. 2022;233:109356. 35286849 10.1016/j.drugalcdep.2022.109356 PMC9750500

[14] Sessa B, Higbed L, O’Brien S, Durant C, Sakal C, Titheradge D, et al. First study of safety and tolerability of 3,4-methylenedioxymethamphetamine-assisted psychotherapy in patients with alcohol use disorder. J Psychopharmacol. 2021;35:375–83. 33601929 10.1177/0269881121991792

[15] Farre M, Tomillero A, Perez-Mana C, Yubero S, Papaseit E, Roset PN, et al. Human pharmacology of 3,4-methylenedioxymethamphetamine (MDMA, ecstasy) after repeated doses taken 4 h apart. Eur Neuropsychopharmacol. 2015;25:1637–49. 26073279 10.1016/j.euroneuro.2015.05.007

[16] Hysek CM, Simmler LD, Schillinger N, Meyer N, Schmid Y, Donzelli M, et al. Pharmacokinetic and pharmacodynamic effects of methylphenidate and MDMA administered alone and in combination. Int J Neuropsychopharmacol. 2014;17:371–81. 24103254 10.1017/S1461145713001132

[17] Hysek CM, Brugger R, Simmler LD, Bruggisser M, Donzelli M, Grouzmann E, et al. Effects of the α_2_-adrenergic agonist clonidine on the pharmacodynamics and pharmacokinetics of 3,4-methylenedioxymethamphetamine in healthy volunteers. J Pharmacol Exp Ther. 2012;340:286–94. 22034656 10.1124/jpet.111.188425

[18] Peiro AM, Farre M, Roset PN, Carbo M, Pujadas M, Torrens M, et al. Human pharmacology of 3,4-methylenedioxymethamphetamine (MDMA, ecstasy) after repeated doses taken 2 h apart. Psychopharmacology (Berl). 2013;225:883–93. 23142957 10.1007/s00213-012-2894-7

[19] Farre M, de la Torre R, Mathuna BO, Roset PN, Peiro AM, Torrens M, et al. Repeated doses administration of MDMA in humans: pharmacological effects and pharmacokinetics. Psychopharmacology (Berl). 2004;173:364–75. 15071716 10.1007/s00213-004-1789-7

[20] Janke W, Debus G Die Eigenschaftswörterliste. Hogrefe: Göttingen; 1978.

[21] Zerssen DV, Petermann F Beschwerden-Liste: Revidierte Fassung. Göttingen: Hogrefe; 2011.

[22] Schmitz N, Kruse J, Tress W. Psychometric properties of the General Health Questionnaire (GHQ-12) in a German primary care sample. Acta Psychiatr Scand. 1999;100:462–8. 10626926 10.1111/j.1600-0447.1999.tb10898.x

[23] Lang G, Bachinger A. Validation of the German Warwick-Edinburgh Mental Well-Being Scale (WEMWBS) in a community-based sample of adults in Austria: a bi-factor modelling approach. J Pubic Health. 2017;25:135–46.

[24] Dieck A, Morin CM, Backhaus J. A German version of the Insomnia Severity Index. Somnologie. 2018;22:27–35.

[25] Luethi D, Rudin D, Straumann I, Thomann J, Avedisian I, Liechti ME, et al. Derivatization-free determination of chiral plasma pharmacokinetics of MDMA and its enantiomers. J Chromatogr B Analyt Technol Biomed Life Sci. 2024;1238:124123. 38615429 10.1016/j.jchromb.2024.124123

[26] Sessa B, Higbed L, Nutt D. A review of 3,4-methylenedioxymethamphetamine (MDMA)-assisted psychotherapy. Front Psychiatry. 2019;10:138. 30949077 10.3389/fpsyt.2019.00138 PMC6435835

[27] Danforth AL, Struble CM, Yazar-Klosinski B, Grob CS. MDMA-assisted therapy: a new treatment model for social anxiety in autistic adults. Prog Neuropsychopharmacol Biol Psychiatry. 2016;64:237–49. 25818246 10.1016/j.pnpbp.2015.03.011

[28] Rudin D, Liechti ME, Luethi D. Molecular and clinical aspects of potential neurotoxicity induced by new psychoactive stimulants and psychedelics. Exp Neurol. 2021;343:113778. 34090893 10.1016/j.expneurol.2021.113778

[29] Baumann MH, Rothman RB. Neural and cardiac toxicities associated with 3,4-methylenedioxymethamphetamine (MDMA). Int Rev Neurobiol. 2009;88:257–96. 19897081 10.1016/S0074-7742(09)88010-0 PMC3153986

[30] Rouaud A, Calder AE, Hasler G. Microdosing psychedelics and the risk of cardiac fibrosis and valvulopathy: comparison to known cardiotoxins. J Psychopharmacol. 2024;38:217–24. 38214279 10.1177/02698811231225609 PMC10944580

[31] Tagen M, Mantuani D, van Heerden L, Holstein A, Klumpers LE, Knowles R. The risk of chronic psychedelic and MDMA microdosing for valvular heart disease. J Psychopharmacol. 2023;37:876–90. 37572027 10.1177/02698811231190865

[32] Luethi D, Liechti ME, Krahenbuhl S. Mechanisms of hepatocellular toxicity associated with new psychoactive synthetic cathinones. Toxicology. 2017;387:57–66. 28645576 10.1016/j.tox.2017.06.004

[33] Liechti ME. Effects of MDMA on body temperature in humans. Temperature. 2014;1:179–87. 10.4161/23328940.2014.955433 PMC5008716 27626046

[34] Schutte JK, Schafer U, Becker S, Oldewurtel C, Starosse A, Singler P, et al. 3,4-Methylenedioxymethamphetamine induces a hyperthermic and hypermetabolic crisis in pigs with and without a genetic disposition for malignant hyperthermia. Eur J Anaesthesiol. 2013;30:29–37. 23138574 10.1097/EJA.0b013e32835a1127

[35] Vizeli P, Liechti ME. Safety pharmacology of acute MDMA administration in healthy subjects. J Psychopharmacol. 2017;31:576–88. 28443695 10.1177/0269881117691569

[36] Verheyden SL, Hadfield J, Calin T, Curran HV. Sub-acute effects of MDMA (±3,4-methylenedioxymethamphetamine, “ecstasy”) on mood: evidence of gender differences. Psychopharmacology (Berl). 2002;161:23–31. 11967627 10.1007/s00213-001-0995-9

[37] Sessa B, Aday JS, O’Brien S, Curran HV, Measham F, Higbed L, et al. Debunking the myth of “Blue Mondays”: no evidence of affect drop after taking clinical MDMA. J Psychopharmacol. 2022;36:360–67. 34894842 10.1177/02698811211055809

[38] Parrott AC, Lasky J. Ecstasy (MDMA) effects upon mood and cognition: before, during and after a Saturday night dance. Psychopharmacology (Berl). 1998;139:261–8. 9784083 10.1007/s002130050714

[39] Pirona A, Morgan MJ. An investigation of the subacute effects of ecstasy on neuropsychological performance, sleep and mood in regular ecstasy users. J Psychopharmacol. 2010;24:175–85. 19351799 10.1177/0269881109102780

[40] Trittibach P, Frueh BE, Goldblum D. Bilateral angle-closure glaucoma after combined consumption of “ecstasy” and marijuana. Am J Emerg Med. 2005;23:813–4. 16182995 10.1016/j.ajem.2005.04.005

[41] Kumar RS, Grigg J, Farinelli AC. Ecstasy induced acute bilateral angle closure and transient myopia. Br J Ophthalmol. 2007;91:693–5. 17446512 10.1136/bjo.2006.099986 PMC1954731

