# Supplementary material for: Comparison of acute effects of 3,4-methylenedioxymethamphetamine (MDMA) with and without a supplemental booster dose in healthy participants: a double-blind, randomized, placebo-controlled, crossover study
Source: Transl Psychiatry. 2026 Jun 4;16:402. doi: 10.1038/s41398-026-04148-6 (PMC13451183; doi:10.1038/s41398-026-04148-6)
Supplement: Supplementary file 1 [file 41398_2026_4148_MOESM1_ESM.pdf]

ONLINE SUPPLEMENTARY MATERIAL

SUPPLEMENTARY METHODS AND RESULTS

Comparison of acute effects of 3,4-methylenedioxymethamphetamine (MDMA) with and without a supplemental booster dose in healthy participants: a double-blind, randomized, placebo-controlled, crossover study

Corresponding author:

Matthias E. Liechti, MD
University Hospital Basel, Department of Clinical Pharmacology and Toxicology
Schanzenstrasse 55
Basel, CH-4031, Switzerland
E-mail: matthias.liechti@usb.ch; Phone: +41 61 328 68 68

Index

1. Supplementary Methods and Materials 3
   - 1.1 Inclusion and exclusion criteria 3
   - 1.2 Drug use/abuse 3
   - 1.3 Table S1 Schedule of events during sessions 4
   - 1.4 Outcome measures 5
     - 1.4.1 Autonomic effects 5
     - 1.4.2 Visual analog scales (VASs) 5
     - 1.4.3 Adjective Mood Rating Scale (AMRS) 5
     - 1.4.4 List of complaints (LC) 5
     - 1.4.5 General Health Questionnaire (GHQ-12) 5
     - 1.4.6 Warwick-Edinburgh Mental Well-Being Scale (WEMWBS) 5
     - 1.4.7 Insomnia Severity Index (ISI) 6
   - 1.5 Handling of blood samples 6
     - 1.5.1 Plasma concentrations of Oxytocin 6
     - 1.5.2 Plasma concentrations of Neurophysin I 6
     - 1.5.3 Routine blood samples 6
   - 1.6 Pharmacokinetic analyses 6
2. Supplementary results 8
   - 2.1 Table S2: Demographics of participants at screening 8
   - 2.2 Table S3: Blinding 8
   - 2.3 Acute effects 9
     - 2.3.1 Table S4 Visual Analog Scales 9
     - 2.3.2 Figure S1 Visual Analog Scales 10
     - 2.3.3 Figure S2 Hysteresis plot illustrating acute tolerance 11
     - 2.3.4 Table S5: Adjective Mood Rating Scale 12
     - 2.3.5 Figure S3 Main scales of the Adjective Mood Rating Scale 13
   - 2.4 Subacute and adverse effects 14
     - 2.4.1 Figure S4 Box plots of the LC, GHQ-12, WEMWBS, and ISI 14
     - 2.4.2 Table S6: Intensity-graded items of the list of complaints 15
     - 2.4.3 Table S7: Frequency-graded items of the list of complaints 16
     - 2.4.4 Table S8: Total score of the LC according to sex, body weight and age 17
     - 2.4.5 Table S9: LC scores for the two dropout participants 18
   - 2.5 Endocrine effects 18
     - 2.5.1 Figure S5: Correlation of oxytocin and neurophysin I 18
     - 2.5.2 Table S10: Mean plasma concentrations of oxytocin and neurophysin I over time 19
   - 2.6 Autonomic measures 20
     - 2.6.1 Table S11: Vital parameters 20
     - 2.6.2 Figure S6 Vital parameters over time 21
     - 2.6.3 Table S12: Blood chemistry and cell counts before and after the study 22
   - 2.7 Pharmacokinetics 22
     - 2.7.1 Figure S7 Mean plasma concentrations of MDMA and its metabolites over time 23
3. Literature 24

1. Supplementary Methods and Materials

1.1 Inclusion and exclusion criteria

Inclusion criteria

1) Age between 18 and 65 years.
2) Good understanding of the German language.
3) Understanding the procedures and the risks that are associated with the study.
4) Participants must be willing to adhere to the protocol and sign the consent form.
5) Participants must be willing to refrain from taking illicit psychoactive substances during the study.
6) Participants must be willing to drink only alcohol-free liquids and no coffee, black or green tea, or energy drinks after midnight of the evening before the study session, as well as during the study day.
7) Participants must be willing not to drive a traffic vehicle or to operate machines within 48h after substance administration.
8) Willing to use effective birth control throughout study participation.
9) Body mass index between 18-29 kg/m^2.

Exclusion criteria

1) Relevant chronic or acute medical condition.
2) Current or previous major psychiatric disorder.
3) Psychotic disorder in first-degree relatives, not including psychotic disorders secondary to an apparent medical reason, e.g. brain injury, dementia, or lesions of the brain.
4) Hypertension (SBP>140/90 mmHg) or hypotension (SBP<85 mmHg).
5) Previous MDMA use more than 20 times or any time within the previous month.
6) Pregnant or nursing women.
7) Participation in another clinical trial (currently or within the last 30 days).
8) Use of medications that may interfere with the effects of the study medications.
9) Tobacco smoking (>10 cigarettes/day).
10) Consumption of alcoholic drinks (>15 drinks/week).

1.2 Drug use/abuse

People interested in participating in the study with past/current excessive substance use or misuse were excluded by the psychological assessment at the screening visit due to either suspected major psychiatric disorder or suspected noncompliance. Cannabis products with < 1% Δ^9-tetrahydrocannabinol (THC) content are legally and commercially available in Switzerland, yet they may result in positive findings in urinary drug tests. Hence, cannabis consumption within the last two months before the screening visit was not an exclusion criterion for this study. Participants were asked to refrain from any illicit drug use including legal cannabis products during the study. Current drug use was tested with urine drug tests at the screening visit and unannounced at the beginning of the dosing sessions. We used *Accu-Tell® Multi-Drug Rapid Test Panel (Urine)*, a rapid chromatographic immunoassay for the qualitative detection of amphetamines, benzodiazepines, cocaine, methadone, morphine, and THC. There were no cases of any positive drug urine tests at the screening or dosing sessions.
A threshold of 10 cigarettes/day was set because smoking was not allowed during the study days and persons smoking more than that have previously reported difficulties remaining abstinent for one day.

1.3 Table S1 Schedule of events during sessions

Relative Time (h)	-0.5	0	0.25	0.5	0.75	1	1.25	1.5	1.75	2	2.25	2.5	2.75	3	3.25	3.5	3.75	4	4.25	4.5
**Substance administration**		1								2										
**Psychometrics**																				
VAS short			1		2		3		4		5		6		7		8		9	
VAS long		0		1		2		3		4		5		6		7		8		9
AMRS	0									1								2		
B-LR	0																			
GHQ-12, SPANE, ISI, WEMBS	0																			
**Blood sampling**																				
Substance concentrations	0			1		2		3		4		5		6		7		8		9
Oxytocin	0									1								2		
**Autonomic measures**																				
BP, HR, BT	0			1		2		3		4		5		6		7		8		9

Relative Time (h)	4.75	5	5.25	5.5	5.75	6	6.25	6.5	6.75	7	7.25	7.5	7.75	8	8.25	8.5	8.75	9	72	168
**Substance administration**																				
**Psychometrics**																				
VAS short	10		11		12		13	14	15		16	17	18		19	20	21			
VAS long		10		11		12				13				14				15		
AMRS						3												4		
B-LR																		1	2	3
GHQ-12, SPANE, ISI, WEMBS																			1	2
**Blood sampling**																				
Substance concentrations		10		11		12				13				14				15		
Oxytocin						3														
**Autonomic measures**																				
BP, HR, BT		10		11		12				13				14				15		

visual analog scales (VAS), adjective mood rating scale (AMRS), general health questionnaire (GHQ-12), Scale of Positive and Negative Experience (SPANE), Warwick-Edingburgh Mental Well-being scale (WEMWBS), Insomnia severity Index (ISI), Beschwerden-Liste (B-LR), blood pressure (BP), heart rate (HR), body temperature (BT)

1.4 Study procedures

Participants were served a standardized breakfast before dosing. Around noon they were offered a menu or small foods such as sandwiches or fruits. They could also eat their menu later as appetite was often suppressed during the acute phase of MDMA.

1.5 Outcome measures

1.5.1 Autonomic effects

Heart rate, systolic blood pressure, and diastolic blood pressure were measured using a Welch Allyn Connex Spot Monitor (Welch Allyn, Inc., 4341 State Street Road, Skaneateles Falls, NY USA) in the dominant arm after a resting time of 5–10 min, with the participant sitting in bed with the back supported. Measures were taken twice per time point with an interval of 1 min, and the average of both measurements was used for analysis. Between measurements, participants were allowed to engage in nonstrenuous activities. Core (tympanic) temperature was also assessed using a Welch Allyn Connex Spot Monitor ear thermometer. The temperature of the room was measured three times during each session. Mean ± SD room temperature was 24 ± 1.6 °C.

1.5.2 Visual analog scales (VASs)

Non-compartmental analyses were applied to all VASs. Maximal effects (E_{max}) were obtained directly from the observed data. Area under the effect curve (AUEC) and effect duration were calculated in Phoenix WinNonlin 8.4 (Certara, Princeton, NJ, USA) using the drug effect module and linear trapezoidal methods with a threshold of 10% for the estimation of effect duration.

1.5.3 Adjective Mood Rating Scale (AMRS)

The Adjective Mood Rating Scale (AMRS or EWL60S) is a 60-item Likert scale that allows repeated assessment of mood in 6 dimensions: Activation, inactivation, well-being, anxiety/depressed mood, extra- and introversion, and emotional excitability. The German EWL60S version was used [1]. The AMRS consists of subscales measuring "activation", "positive mood", "extraversion", "introversion", "inactivation", and "emotional excitability. The scale has previously been used to evaluate effects of many other psychoactive substances.

1.5.4 List of complaints (LC)

Originally developed in 1976 [2], the Beschwerden-Liste (list of complaints, LC) is a self-reporting tool to assess physical and general discomfort. We used the revised version of 2011 [3]. It consists of 40 individual items with a four-point intensity-scoring ranging from 0 (not at all) to 3 (strong). We added 10 items based on frequently reported complaints in previous MDMA research trials, resulting in a total of 50 items. The intensity-graded complaints were added to a total symptom score, which was compared between conditions and over time. Additionally, single item occurrences were summed for each condition and timepoint if the given item was rated >0.
The LC was administered before (baseline, 0 h), at the end of the dosing session (acute, 0–9 h), as well as 3 and 7 days (subacute, 0–3 and 3–7 days, respectively) after each session assessing any complaints during the time interval since the last assessment.

1.5.5 General Health Questionnaire (GHQ-12)

The General Health Questionnaire was originally developed in 1972 as a screening tool to detect psychiatric illness [4]. Originating from the 60-item GHQ, several shorter versions have been developed and validated. The GHQ-12 has been widely used in different clinical settings, due to its brevity and its relatively good psychometric properties. It consists of a 12-item questionnaire with a four-point response scale assessing general psychological discomfort. Although the GHQ-12 was designed as a unidimensional scale, several studies revealed two- or three-factor solutions, and different scoring methods have been used [5]. We used the German version [6] with a unidimensional scale and a four-point Likert-Rating and compared total scores at different timepoints between groups. The score ranges from 0 to 36, with higher values indicating more severe complaints.

1.5.6 Warwick-Edinburgh Mental Well-Being Scale (WEMWBS)

The Warwick-Edinburgh Mental Well-Being Scale is a 14-item questionnaire, that covers different concepts associated with positive mental health, including both hedonic and eudaimonic aspects, positive affect, satisfying interpersonal relationships and positive functioning [7]. For each item an ordinal

five-point frequency answer format, ranging from 1 = “none of the time” to 5 = “all of the time” is applied. We used the validated German translation of the WEMWBS [8]. Total scores range from 14 to 70 points, with higher values indicating higher levels of well-being.

1.5.7 Insomnia Severity Index (ISI)

The Insomnia Severity Index [9] is a self-report instrument to assess subjective sleep quality, screen for insomnia, and evaluate treatment response. It consists of seven items scored on a five-point Likert-scale. Total scores range from 0 to 28, with higher values indicating worse sleep quality. We used the validated German version [10].

1.6 Handling of blood samples

Plasma concentrations of MDMA

Blood samples were collected into lithium-heparin tubes. After collection, the blood was centrifuged at 1.4 rcf and 4 °C for 10 min, and the plasma was stored at -80 °C until analysis. Plasma concentrations of MDMA, MDA, and HMMA were analyzed using a fully validated ultra-high-performance liquid chromatography tandem mass spectrometry method [11]. HMMA concentration was determined after enzymatic deglucuronidation.

1.6.1 Plasma concentrations of Oxytocin

Blood samples were collected into EDTA tubes. After collection, the blood was centrifuged at 1.4 rcf and 4 °C for 10 min, and the plasma was stored at -80 °C until analysis. Oxytocin concentrations in plasma were measured using an oxytocin enzyme-linked immunosorbent assay kit (ENZO Life Sciences, Ann Arbor, MI, United States) according to the manufacturer’s protocol. Plasma was diluted in a 2:1 ratio in 1.5% TFA-H_2O and centrifuged at 17,100 × *g* for 15 min at 4 °C. The supernatant was collected and then loaded onto an Oasis PRiME HLB 96-well plate with 30 mg sorbent (Waters, Milford, MA, United States) and washed with 0.1% TFA-H_2O. The sample was then eluted with 95% acetonitrile + 5% of a 0.1% TFA-H_2O solution. The elute was collected and dried in an Eppendorf centrifugal vacuum concentrator (model no. 5301, Eppendorf AG, Hamburg, Germany). For the measurements, the residue was reconstituted in assay buffer. The reconstituted samples, standards, and controls were plated on a goat anti-rabbit immunoglobulin G microtiter plate and incubated with oxytocin conjugate at 4 °C overnight. The plate was then washed, and the substrate was added. The reaction was stopped after 1 h, and the plate was read at 405 nm. Oxytocin concentrations were determined from a standard curve that was calculated from a four-parameter logistics curve fit.

1.6.2 Plasma concentrations of Neurophysin I

Neurophysin I was measured in plasma samples using the Oxytocin-neurophysin I prepropeptide *in vitro* SimpleStep ELISA kit (Abcam, Cambridge, UK) according to the manufacturer’s protocol. Plasma samples were obtained at the same timepoints as the oxytocin measurements and were collected in EDTA tubes. After collection, the blood was centrifuged at 1.4 rcf and 4 °C for 10 min, and the plasma was stored at -80 °C until analysis.

1.6.3 Routine blood samples

Blood chemistry (creatinine, alanine aminotransferase, glomerular filtration rate) and blood cell count tests were performed at the screening visit at the start of the study and at the EOS visit. The analyses were performed using standard assays according to Good Laboratory Practice by the Laboratory Medicine Department of the hospital. The glomerular filtration rate was determined by the CKD-EPI Equation using plasma creatinine concentrations, age, and sex of the participant.

1.7 Pharmacokinetic analyses

The pharmacokinetic parameters of MDMA, MDA and HMMA were calculated using non-compartmental analyses in Phoenix WinNonlin 8.4 (Certara, Princeton, NJ, USA). Peak plasma concentration (C_{max}) and time to C_{max} (t_{max}) were obtained directly from the observed data. The terminal elimination rate constant (λ_z) to calculate half-life ($t_{1/2}$) was estimated by log-linear regression after semilogarithmic transformation of the data using at least three data points of the linear elimination phase of the concentration-time curve. An adjusted r^2 of 0.8 of this regression was determined as minimal acceptance criterion for λ_z. In profiles with higher r^2-values, pharmacokinetic parameters depending on λ_z were excluded from the summary statistics. The area under the concentration-time curve (AUC) from 0 h to the last postdosing time point ($AUC_{0\text{-}9}$) was computed employing the linear-up log-down method. Pharmacologic parameters were summarized as geometric means with respective 95% confidence

intervals (CI). The MDMA and Booster conditions were compared by calculating geometric mean ratios with the respective 90% CI for C_{max} and AUC_{0-9}.

2. Supplementary results

2.1 Table S2: Demographics of participants at screening

	**All included participants**	**Without dropouts**
Number of participants, *n*	25	23
Sex, female/male, *n*	13/12	11/12
Age, years	36 ± 13	35 ± 13
range	20–56	20–65
Body weight, kg	66 ± 11	67 ± 12
range	51–95	51–95
BMI, kg/m^2	22 ± 2.8	22 ± 2.9
Any previous MDMA use, *n* (%)	10 (40)	10 (43)
amount of previous MDMA use in users	5.3 ± 3.8	5.3 ± 3.8
range of previous MDMA use in users	1–14	1–14
Smoking, *n* (%)	4 (16)	4 (17)
Range of cigarettes/day in smokers	1–5	1–5
Regular alcohol consumption, *n* (%)	23 (92)	21 (91)
amount of standard drinks/week in drinkers	2.9 ± 2.9	2.8 ± 2.9
Any previous cannabis use, *n* (%)	23 (92)	21 (92)
amount of previous use in users	166 ± 286	181 ± 295
Any previous hallucinogenic use, *n* (%)	15 (60)	14 (61)
amount of previous use in users	6.5 ± 5.4	6.8 ± 5.4
Any previous sedative use, *n* (%)	4 (16)	4 (17)
amount of previous use in users	5.5 ± 4.4	5.5 ± 4.4
Any previous opioid use, *n* (%)	3 (12)	3 (13)
amount of previous use in users	1.3 ± 0.6	1.3 ± 0.6
Any previous stimulant use, *n* (%)	9 (36)	9 (39)
amount of previous use in users	11 ± 16	11 ± 16

Values are shown as mean ± standard deviation, minimum–maximum for range, or counts. *n*= number of participants.

2.2 Table S3: Blinding

	**correctly guessed allocation**	
	after session	after study
**Actual allocation**		
Placebo	23/23	23/23
MDMA	20/23	20/23
Booster	15/23	20/23

Participants were asked to guess the condition after each session and at the end of study visit. Shown are correct/total guesses.

2.3 Acute effects

2.3.1 Table S4 Visual Analog Scales

Condition		Booster			MDMA			Placebo			$F_{2, 66}$	*p*	Booster-MDMA	Booster-Placebo	MDMA-Placebo
		mean	±	SD	mean	±	SD	mean	±	SD					
**Unidirectional scales (0%–100%)**															
Drug high	E_{max}	79	±	23	76	±	25	3.1	±	12	153	<0.001	NS	***	***
	AUEC	332	±	182	265	±	187	14	±	65	48.6	<0.001	*	***	***
Stimulated	E_{max}	72	±	32	68	±	32	3.4	±	13	80	<0.001	NS	***	***
	AUEC	292	±	161	231	±	171	9.8	±	44	39.7	<0.001	NS	***	***
Drug liking	E_{max}	84	±	20	84	±	20	8.0	±	24	118	<0.001	NS	***	***
	AUEC	410	±	184	333	±	188	23	±	95	68.5	<0.001	*	***	***
**Bidirectional scales (-50% to +50%)**															
Be alone	E_{max}	8.8	±	13	9.4	±	16	0.3	±	1.5	4.9	<0.05	NS	*	*
	E_{min}	-35	±	20	32	±	22	-3.3	±	11	26.4	<0.001	NS	***	***
	AUEC	-146	±	149	-97	±	133	-5.4	±	21	11.9	<0.001	NS	***	**
Feeling close to others	E_{max}	31	±	18	28	±	18	1.1	±	4.6	45.8	<0.001	NS	***	***
	E_{min}	-3.7	±	8.9	-8.7	±	16	-0.5	±	2.3	4.52	<0.05	NS	NS	NS
	AUEC	129	±	114	70	±	117	4.3	±	20	17.2	<0.001	**	***	*
Emotional	E_{max}	25	±	18	26	±	19	1.0	±	5.0	32.9	<0.001	NS	***	***
	E_{min}	-4.2	±	11	-5.6	±	12	-2.2	±	10	0.603	0.552	NS	NS	NS
	AUEC	82	±	98	69	±	105	3.2	±	21	9.37	<0.001	NS	**	*
Happy	E_{max}	35	±	14	34	±	15	1.1	±	4.8	74.6	<0.001	NS	***	***
	E_{min}	-3.9	±	7.5	-2.7	±	5.3	0.0	±	0.0	4.03	<0.05	NS	NS	NS
	AUEC	130	±	98	97	±	93	4.8	±	23	25.6	<0.001	NS	***	***

SD, standard deviation; AUEC, area under the effect-time curve; E_{max}, maximal effects; E_{min}, minimal effects. The MDMA condition consisted of a MDMA single dose (120 mg), the Booster condition was a 180 mg split dose (120 mg followed by 60 mg after 2 hours). AUECs for bidirectional VAS were calculated by subtracting the AUEC below baseline from the AUEC above baseline. Repeated Measures Analysis of Variance (ANOVAs) followed by Tukey post hoc tests were used. **p*< 0.05, ***p*< 0.01, ****p*<0.001; NS, not significant, *n*=23.

2.3.2 Figure S1 Visual Analog Scales

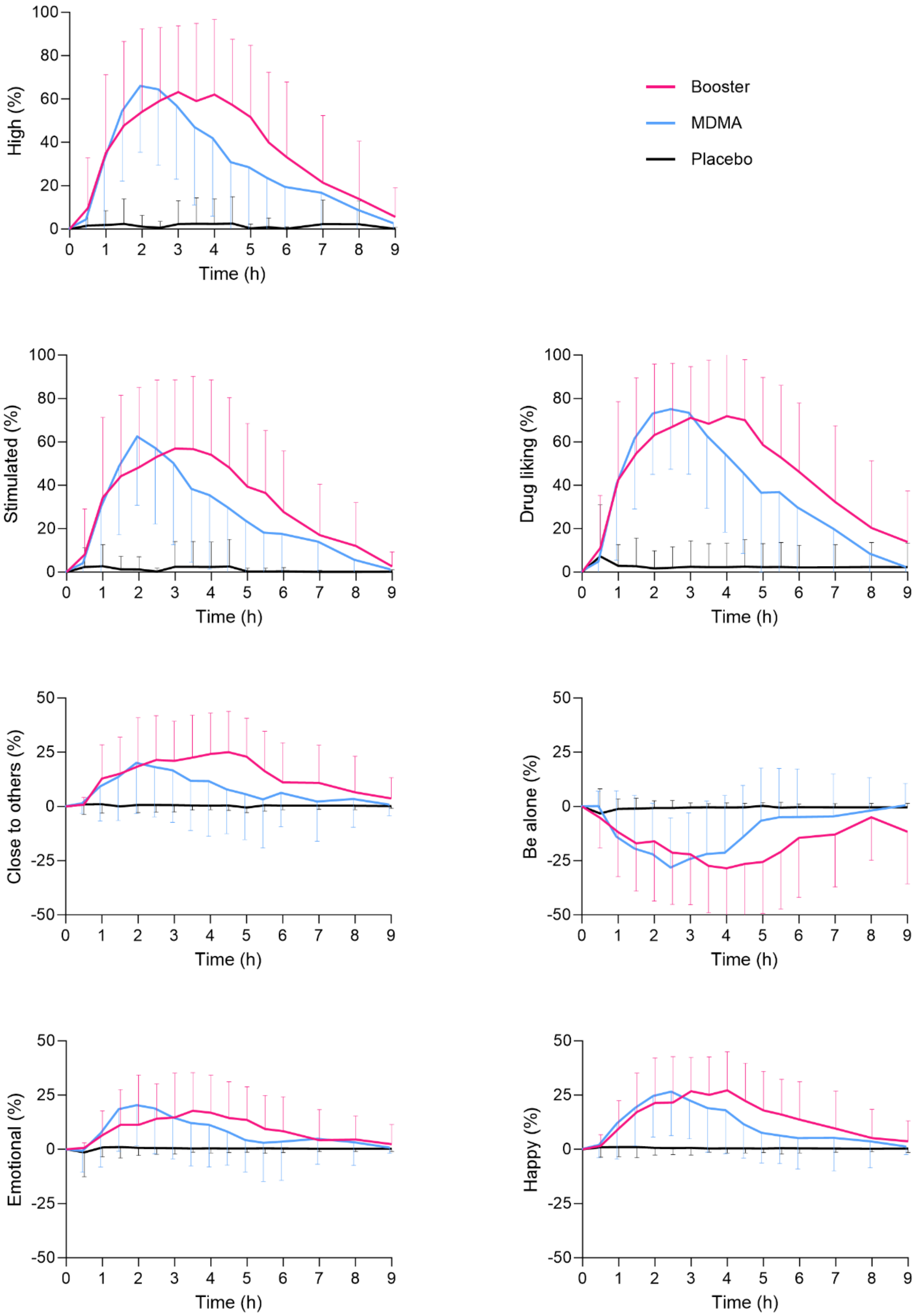

Figure S1 Results from single-item visual analog scales over time. Data are mean scores (%) with standard deviation as error bars. *n*=23.

2.3.3 Figure S2 Hysteresis plot illustrating acute tolerance

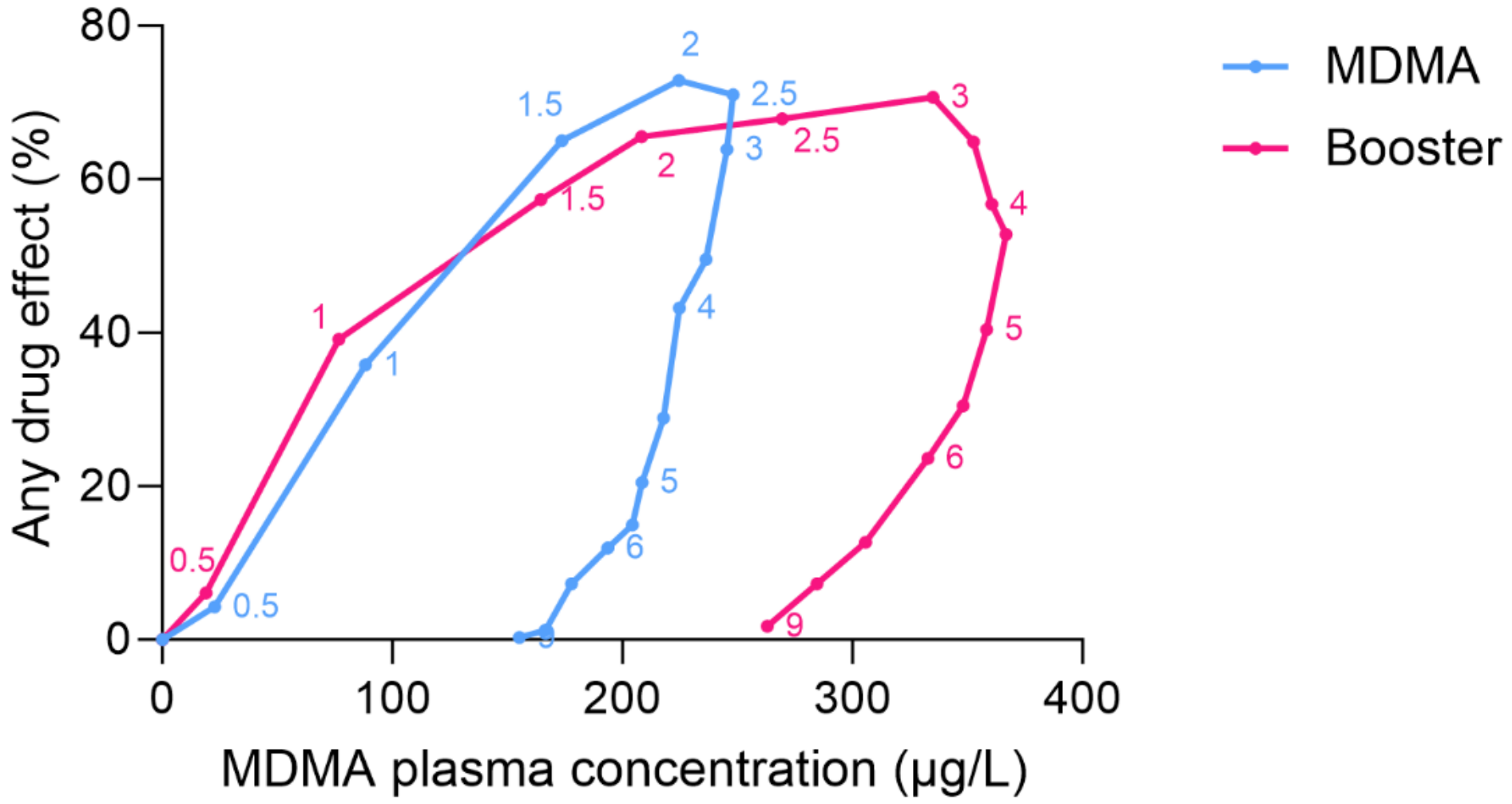

Figure S2 Hysteresis plot showing acute effects measured by the visual analog scale item "any drug effect" and MDMA plasma concentrations. The respective time points in hours are indicated directly on the curves. Data are mean, *n*=23.

2.3.4 Table S5: Adjective Mood Rating Scale

	Time point	Booster	MDMA	Placebo	$F_{2, 44}$	*p*	Booster - MDMA	Booster - Placebo	MDMA - Placebo
	h	mean ± SD	mean ± SD	mean ± SD					
**AMRS main scales**									
Activity and performance									
	2	-0.30 ± 5.0	0.48 ± 5.6	-2.4 ± 2.6	3.06	0.057	NS	NS	NS
	4	1.3 ± 4.7	-0.57 ± 5.1	-2.9 ± 3.2	4.86	<0.05	NS	*	NS
	6	-1.6 ± 4.5	-2.4 ± 5.3	-3.3 ± 3.1	0.93	0.403	NS	NS	NS
	9	-2.9 ± 3.2	-2.5 ± 3.7	-4.0 ± 3.2	1.14	0.329	NS	NS	NS
General inactivity									
	2	0.0 ± 4.3	1.7 ± 5.4	0.70 ± 3.5	0.79	0.459	NS	NS	NS
	4	-0.2 ± 3.7	3.7 ± 6.7	0.52 ± 4.2	3.97	<0.05	NS	NS	NS
	6	2.3 ± 5.0	6.2 ± 5.8	0.78 ± 3.8	8.84	<0.001	**	NS	**
	9	4.5 ± 6.2	4.3 ± 5.6	0.96 ± 3.6	3.88	<0.05	NS	NS	NS
Well-being									
	2	1.8 ± 5.0	3.7 ± 4.6	-1.1 ± 2.8	7.58	<0.01	NS	NS	**
	4	4.2 ± 4.5	0.17 ± 5.4	-1.3 ± 2.9	11.31	<0.001	*	***	NS
	6	1.0 ± 4.9	-1.9 ± 4.5	-2.2 ± 3.1	5.68	<0.01	*	**	NS
	9	-1.9 ± 3.1	-1.9 ± 4.4	-2.1 ± 3.0	0.03	0.969	NS	NS	NS
Extroversion/introversion									
	2	0.57 ± 3.8	1.3 ± 3.7	-0.65 ± 2.0	2.52	0.092	NS	NS	NS
	4	2.5 ± 3.8	-0.30 ± 3.9	-1.6 ± 2.2	8.59	<0.001	NS	***	NS
	6	-0.83 ± 4.2	-2.0 ± 3.6	-1.6 ± 2.0	1.04	0.361	NS	NS	NS
	9	-2.5 ± 2.6	-1.4 ± 3.0	-1.5 ± 2.6	1.19	0.314	NS	NS	NS
Emotional excitation									
	2	1.7 ± 4.0	2.5 ± 3.5	-2.3 ± 2.2	19.07	<0.001	NS	**	***
	4	1.6 ± 3.6	0.87 ± 3.0	-2.6 ± 2.6	16.37	<0.001	NS	***	***
	6	0.39 ± 2.9	-0.35 ± 2.8	-2.7 ± 2.3	9.37	<0.001	NS	**	*
	9	-0.65 ± 2.1	-1.4 ± 1.9	-2.6 ± 2.3	5.61	<0.01	NS	*	NS

AMRS, adjective mood rating scale; MDMA, 3,4-methylenedioxymethamphetamine; SD, standard deviation. Results are mean ± SD difference to baseline (at time = 0) scores. The MDMA condition consisted of a MDMA single dose (120 mg), the Booster condition was a 180 mg split dose (120 mg followed by 60 mg after 2 hours). Repeated Measures Analysis of Variance (ANOVAs), followed by Tukey post hoc tests were used. *p<0.05; **p<0.01; ***p<0.001; NS, not significant, n=23.

2.3.5 Figure S3 Main scales of the Adjective Mood Rating Scale

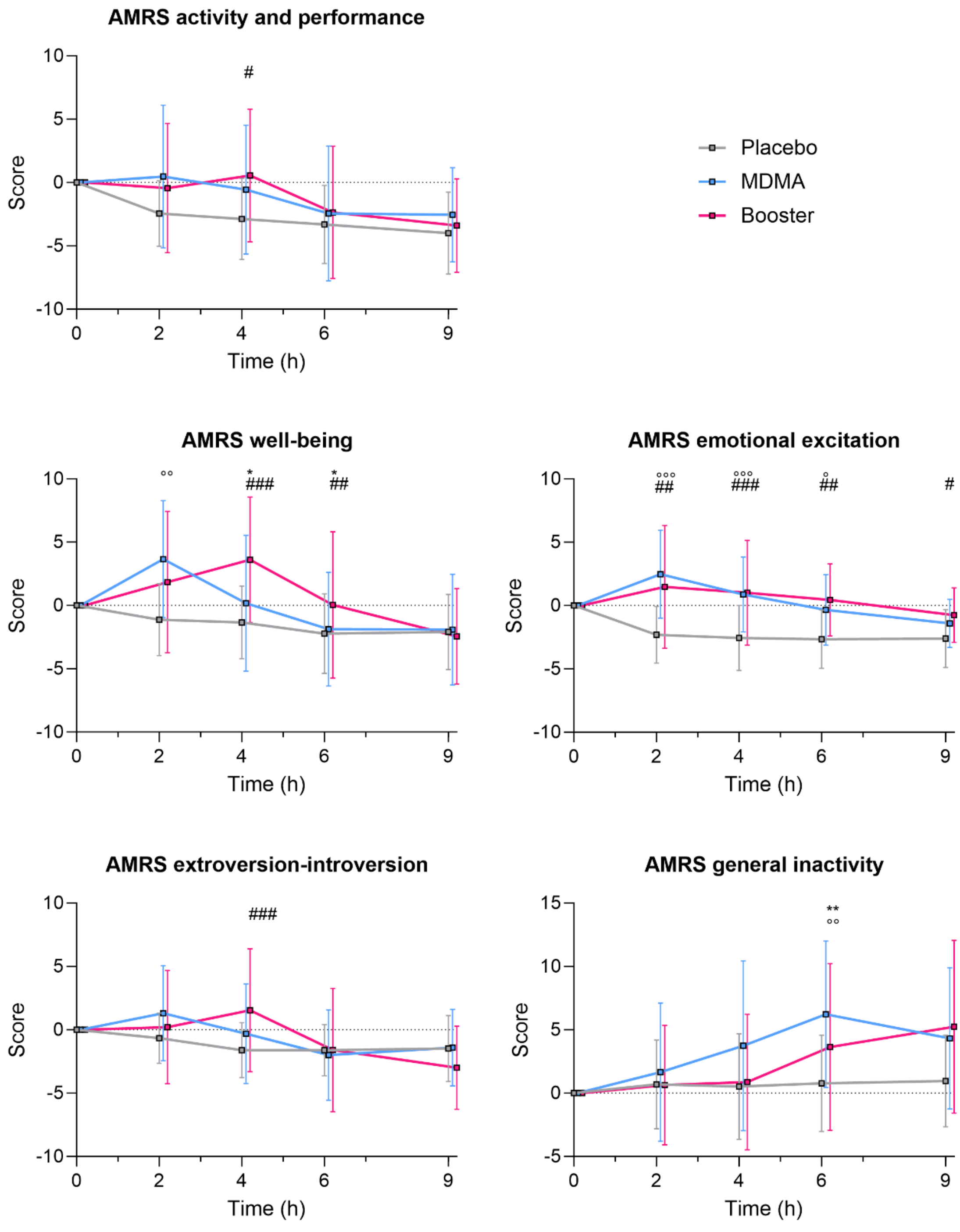

Figure S3 Main scales of the Adjective Mood Rating Scale (AMRS) over time. Data are presented as mean difference to baseline values at different time points (staggered for better readability), with standard deviation as error bars. *$P < 0.05$, **$P < 0.01$, ***$P < 0.001$ Booster compared to MDMA, #$P < 0.05$, ##$P < 0.01$, ###$P < 0.001$ Booster compared to Placebo, °$P < 0.05$, °°$P < 0.01$, °°°$P < 0.001$ MDMA compared to Placebo. *n=23.*

2.4 Subacute and adverse effects

2.4.1 Figure S4 Box plots of the LC, GHQ-12, WEMWBS, and ISI

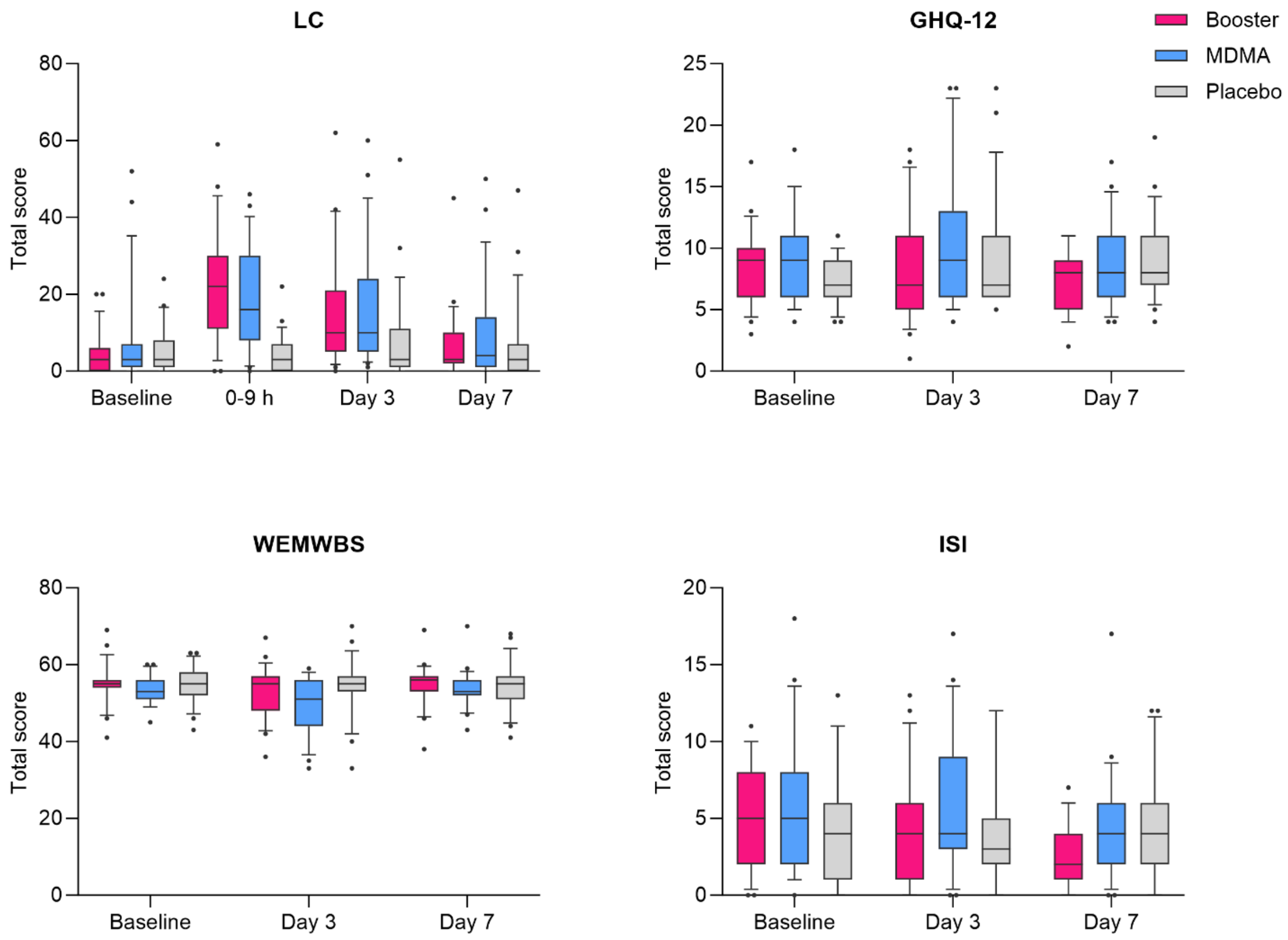

Figure S4 Box plots showing acute and subacute adverse effects. LC, list of complaints; GHQ-12, General Health Questionnaire-12; WEMWBS, Warwick-Edinburgh Mental Well-being Scale; ISI, Insomnia Severity Index. Data are absolute total scores at different time points; whiskers are at the 10th and 90th percentile. *n=23.*

2.4.2 Table S6: Intensity-graded items of the list of complaints

Condition	Booster				MDMA				Placebo			
Time point/interval (h)	0	0–9	9–84	84–168	0	0–9	9–84	84–168	0	0–9	9–84	84–168
**Complaint**												
Bruxism	3	37	11	6	2	23	10	5	4	1	3	4
Tiredness	18	29	25	14	22	30	32	17	22	19	14	12
Lack of appetite	1	29	8	1	3	34	10	4	1	1	1	2
Feeling dull	10	23	21	5	12	16	22	10	5	7	4	7
Dry mouth	4	23	9	2	9	19	7	5	3	2	3	3
Lack of energy	4	22	18	7	8	18	25	13	6	10	6	7
Headache	3	20	16	4	3	23	18	8	4	13	4	6
Hot flashes	2	19	5	2	2	19	4	1	1	1	3	2
Feeling of weakness	4	18	15	5	4	10	15	8	3	2	3	4
Perspiration	2	18	9	2	0	24	6	3	2	0	3	3
Increased need to sleep	3	18	23	8	5	26	26	10	7	9	5	8
Feeling restless	7	16	13	4	9	13	11	11	6	2	11	7
Rapid fatigue	5	16	14	4	8	13	19	10	2	5	4	4
Dizziness	1	15	9	3	1	12	10	7	1	1	2	2
Impaired concentration	4	15	12	6	3	14	16	6	0	4	5	3
Inner tension	7	14	14	4	13	10	10	9	8	2	12	5
Brooding	3	12	16	10	6	15	16	13	5	0	8	10
Restless legs	2	12	7	2	0	7	2	1	5	0	2	3
Heavy legs	0	11	8	4	4	13	9	4	1	2	3	3
Shivering	1	10	7	3	1	7	3	1	1	0	0	1
Freezing	0	9	4	1	4	9	7	1	1	0	4	2
Muscle tension	2	9	7	2	5	8	4	4	3	5	6	3
Nausea	0	8	8	3	0	6	5	4	0	1	2	2
Hypersensitivity to warm	0	8	1	0	1	7	3	1	1	0	0	3
Throat tightness	3	7	2	3	3	2	5	4	4	1	6	1
Insomnia	2	7	7	4	10	2	9	8	4	0	7	7
Decreased libido	4	7	5	3	5	9	8	5	2	3	4	4
Numbness in hands or feet	1	7	1	1	2	2	1	1	0	1	2	2
Impaired balance	0	6	4	1	1	9	4	4	0	1	2	1
Palpitations	0	6	4	3	2	10	1	2	0	0	2	1
Negative thoughts	4	6	7	6	3	4	11	5	1	0	5	5
Vomiting	0	6	0	0	0	3	1	1	0	0	0	0
Abdominal pain	1	6	4	3	1	0	1	1	0	0	1	1
Hypersensitivity to cold	1	5	7	2	5	10	6	0	1	0	2	2
Chest pain	0	4	4	3	0	3	4	3	1	1	3	3
Diarrhea	0	4	3	4	1	0	3	3	1	0	3	1
Dysphagia	2	3	1	1	2	0	1	1	4	2	2	1
Blushing	1	3	0	1	1	1	3	1	1	1	0	1
Muscle cramps	1	3	4	1	1	0	3	1	1	0	0	0
Dyspepsia	0	3	2	3	0	1	2	2	0	0	1	0
Back pain	7	3	2	3	9	7	6	5	9	5	6	5
Dyspnea	1	2	1	2	2	1	5	2	1	0	3	2
Hypersensitivity to certain odors	1	2	3	2	6	2	4	3	1	0	1	1
Irritability	3	1	10	7	2	1	12	3	2	0	7	4
Feeling suffocated	0	1	0	0	1	1	1	0	0	0	1	1
Tendency to cry	0	1	6	3	2	1	4	3	0	0	5	4
Constipation	0	1	2	0	1	0	3	1	1	0	2	0
Decrease in body weight	0	0	1	1	5	0	2	2	1	0	0	2
Intermittent shortness of breath	0	0	0	0	0	0	0	0	0	0	0	0
Anxiety	1	0	3	0	3	4	5	4	1	0	3	3
**Total Score**	119	505	363	159	193	449	395	221	128	102	176	158

Each item was scored using intensity-grading (0–3). Shown are the sums for each item at each condition and each time point and the total scores for each condition and timepoint. The MDMA condition consisted of a MDMA single dose (120 mg), the Booster condition was a 180 mg split dose (120 mg followed by 60 mg after 2 hours). *n*=23.

2.4.3 Table S7: Frequency-graded items of the list of complaints

Condition	Booster				MDMA				Placebo			
Time point/interval (h)	0	0–9	9–84	84–168	0	0–9	9–84	84–168	0	0–9	9–84	84–168
**Complaint**												
Bruxism	3	18	7	3	2	14	6	3	4	1	2	3
Tiredness	14	16	15	12	15	15	18	12	14	13	10	11
Feeling dull	8	15	12	5	9	10	13	9	4	5	4	6
Dry mouth	4	15	7	2	8	14	7	5	3	2	3	3
Lack of appetite	1	14	7	1	2	17	5	3	1	1	1	2
Feeling of weakness	4	13	10	5	3	8	10	5	3	2	2	3
Feeling restless	7	12	9	4	7	11	7	9	6	2	7	6
Lack of energy	4	12	10	7	5	11	15	9	5	8	5	6
Impaired concentration	4	12	8	6	2	11	12	4	0	4	5	3
Dizziness	1	11	7	2	1	8	7	5	1	1	2	2
Headache	3	11	13	4	3	12	10	7	4	9	4	4
Hot flashes	2	11	3	1	2	13	4	1	1	1	2	2
Inner tension	6	11	12	4	10	9	7	8	8	2	8	5
Perspiration	2	10	5	1	0	14	5	3	2	0	2	2
Increased need to sleep	3	10	14	7	4	12	14	8	5	7	4	6
Rapid fatigue	5	10	10	4	6	11	11	7	1	4	2	4
Brooding	3	9	11	9	4	10	10	9	4	0	7	7
Shivering	1	9	5	2	1	7	3	1	1	0	0	1
Heavy legs	0	7	5	3	3	11	6	3	1	2	3	2
Hypersensitivity to warm	0	7	1	0	1	4	3	1	1	0	0	2
Freezing	0	7	3	1	4	5	5	1	1	0	3	2
Restless legs	2	6	5	2	0	5	2	1	4	0	2	3
Impaired balance	0	6	3	1	1	8	3	2	0	1	2	1
Palpitations	0	6	3	2	1	6	1	1	0	0	2	1
Negative thoughts	3	6	6	6	3	4	10	4	1	0	5	5
Abdominal pain	1	6	3	3	1	0	1	1	0	0	1	1
Muscle tension	2	6	5	2	4	6	4	4	3	4	5	3
Nausea	0	5	7	2	0	4	4	3	0	1	2	2
Hypersensitivity to cold	1	5	5	2	4	6	4	0	1	0	1	1
Decreased libido	4	5	5	2	3	6	6	3	2	2	4	4
Numbness in hands or feet	1	5	1	1	1	2	1	1	0	1	2	1
Throat tightness	2	4	2	2	3	2	3	4	2	1	4	1
Insomnia	2	4	5	4	7	2	7	6	3	0	6	5
Vomiting	0	3	0	0	0	2	1	1	0	0	0	0
Dyspepsia	0	3	2	3	0	1	2	2	0	0	1	0
Back pain	5	3	2	3	7	6	4	3	6	3	6	4
Dyspnea	1	2	1	1	2	1	3	2	1	0	2	2
Dysphagia	2	2	1	1	2	0	1	1	2	2	1	1
Chest pain	0	2	2	2	0	2	2	2	1	1	2	2
Blushing	1	2	0	1	1	1	2	1	1	1	0	1
Diarrhea	0	2	1	3	1	0	2	2	1	0	3	1
Muscle cramps	1	2	3	1	1	0	3	1	1	0	0	0
Hypersensitivity to certain odors	1	2	2	2	3	2	3	2	1	0	1	1
Irritability	3	1	10	6	1	1	10	3	2	0	4	4
Feeling suffocated	0	1	0	0	1	1	1	0	0	0	1	1
Tendency to cry	0	1	4	3	2	1	3	2	0	0	3	4
Constipation	0	1	2	0	1	0	2	1	1	0	1	0
Decrease in body weight	0	0	1	1	3	0	2	1	1	0	0	2
Intermittent shortness of breath	0	0	0	0	0	0	0	0	0	0	0	0
Anxiety	1	0	2	0	2	4	5	3	1	0	3	2
**Total Score**	108	331	257	139	147	300	270	170	105	81	140	135

Each item was initially scored intensity-graded (0–3) and values >0 were counted as 1. Shown are the sums for each item at each condition and at each time point and the total scores for each condition and time point. The MDMA condition consisted of a MDMA single dose (120 mg), the Booster condition was a 180 mg split dose (120 mg followed by 60 mg after 2 hours). *n*=23.

2.4.4 Table S8: Total score of the LC according to sex, body weight and age

Condition	Booster		MDMA		Placebo	
	mean ± SD		mean ± SD		mean ± SD	
**Sex**	**female**	**male**	**female**	**male**	**female**	**male**
	n=11	*n*=12	*n*=11	*n*=12	*n*=11	*n*=12
**LC total score**						
Baseline	6.4 ± 7.4	4.1 ± 4.9	13 ± 18	3.9 ± 5.1	9.2 ± 7.6	2.3 ± 2.2
Acute (0–9 h)	33 ± 13	12 ± 7.6	27 ± 14	13 ± 8.8	4.5 ± 4.4	4.4 ± 6.2
Subacute (Days 0–3)	23 ± 19	8.8 ± 6.6	23 ± 20	12 ± 9.8	13 ± 17	2.7 ± 1.4
Subacute (Days 4–7)	11 ± 13	3.5 ± 3.5	15 ± 17	4.4 ± 5.7	11 ± 15	2.7 ± 2.3
**LC difference to baseline score**						
Acute (0–9 h)	27 ± 7.3	7.6 ± 8.6	13 ± 15	9.1 ± 7.8	-4.7 ± 7.2	2.2 ± 5.1
Subacute (Days 0–3)	17 ± 14	4.7 ± 5.5	9.5 ± 12	8.2 ± 12	3.9 ± 15	0.42 ± 2.4
Subacute (Days 4–7)	4.3 ± 8.4	-0.58 ± 5.1	2.0 ± 17	0.50 ± 8.3	2.3 ± 13	0.42 ± 3.5

Condition	Booster		MDMA		Placebo	
	mean ± SD		mean ± SD		mean ± SD	
**Weight, kg**	**<65**	**≥65**	**<65**	**≥65**	**<65**	**≥65**
	n=10	*n*=13	*n*=10	*n*=13	*n*=10	*n*=13
**LC total score**						
Baseline	5.3 ± 6.2	5.1 ± 6.3	9.5 ± 16	7.5 ± 12	7 ± 8.3	4.5 ± 4.6
Acute (0–9 h)	30 ± 14	16 ± 13	22 ± 16	18 ± 11	2.6 ± 3.3	5.8 ± 6.2
Subacute (Days 0–3)	19 ± 15	13 ± 16	18 ± 18	17 ± 16	8.2 ± 9.9	7.2 ± 15
Subacute (Days 4–7)	6.2 ± 6.2	7.5 ± 12	10 ± 14	9.1 ± 14	7.2 ± 9.9	6.6 ± 13
**LC difference to baseline score**						
Acute (0–9 h)	24 ± 9.6	11 ± 12	13 ± 13	10 ± 11	-4.4 ± 7.8	1.4 ± 5.3
Subacute (Days 0–3)	14 ± 13	8.2 ± 12	8.0 ± 12	9.4 ± 12	1.2 ± 9.6	2.8 ± 11
Subacute (Days 4–7)	0.90 ± 3.3	2.4 ± 9.2	0.80 ± 17	1.5 ± 8.2	0.20 ± 8.7	2.2 ± 9.7

Condition	Booster		MDMA		Placebo	
	mean ± SD		mean ± SD		mean ± SD	
**Age, years**	**<30**	**≥30**	**<30**	**≥30**	**<30**	**≥30**
	n=10	*n*=13	*n*=10	*n*=13	*n*=10	*n*=13
**LC total score**						
Baseline	3.7 ± 3.3	6.3 ± 7.3	3.9 ± 6.4	12 ± 16	3.4 ± 3.1	7.2 ± 7.5
Acute (0–9 h)	22 ± 11	22 ± 17	17 ± 12	22 ± 13	2.9 ± 2.8	5.6 ± 6.3
Subacute (Days 0–3)	11 ± 12	19 ± 17	7.8 ± 6.1	24 ± 18	5.7 ± 9.6	9.2 ± 14
Subacute (Days 4–7)	4.4 ± 4.6	8.8 ± 12	3.1 ± 4.0	15 ± 16	4.5 ± 9.1	8.7 ± 12
**LC difference to baseline score**						
Acute (0–9 h)	18 ± 9.7	16 ± 14	13 ± 10	9.9 ± 12	-0.50 ± 2.1	-1.6 ± 8.9
Subacute (Days 0–3)	7.7 ± 9.4	13 ± 13	3.9 ± 3.7	13 ± 14	2.3 ± 8.3	1.9 ± 12
Subacute (Days 4–7)	0.70 ± 3.2	2.5 ± 8.8	-0.80 ± 3.2	2.8 ± 16	1.1 ± 7.9	1.5 ± 9.8

MDMA, 3,4-methylenedioxymethamphetamine; LC, list of complaints; SD, standard deviation. Intensity-graded total scores of the LC were stratified according to sex, age, and weight groups. The MDMA condition consisted of a MDMA single dose (120 mg), the Booster condition was a 180 mg split dose (120 mg followed by 60 mg after 2 hours). *n*= number of participants.

2.4.5 Table S9: LC scores for the two dropout participants

Condition	Booster		
	first dropout	second dropout	mean
	n=1	*n*=1	*n*=2
Sex	female	female	
Weight, kg	61	55	58
Age, years	51	28	40
**LC total score**			
Baseline	39	9	24
Acute (0–9 h)	81	52	67
Subacute (Days 0–3)	100	73	87
Subacute (Days 4–7)	34	29	32
**LC difference to baseline score**			
Acute (0–9 h)	42	43	43
Subacute (Days 0–3)	61	64	63
Subacute (Days 4–7)	28	50	39

MDMA, 3,4-methylenedioxymethamphetamine; LC, list of complaints. Intensity-graded total scores of the LC for both participants that dropped-out after the first session which was the Booster condition in both. The Booster condition consisted of a 180 mg MDMA split dose (120 mg followed by 60 mg after 2 hours).

2.5 Endocrine effects

2.5.1 Figure S5: Correlation of oxytocin and neurophysin I

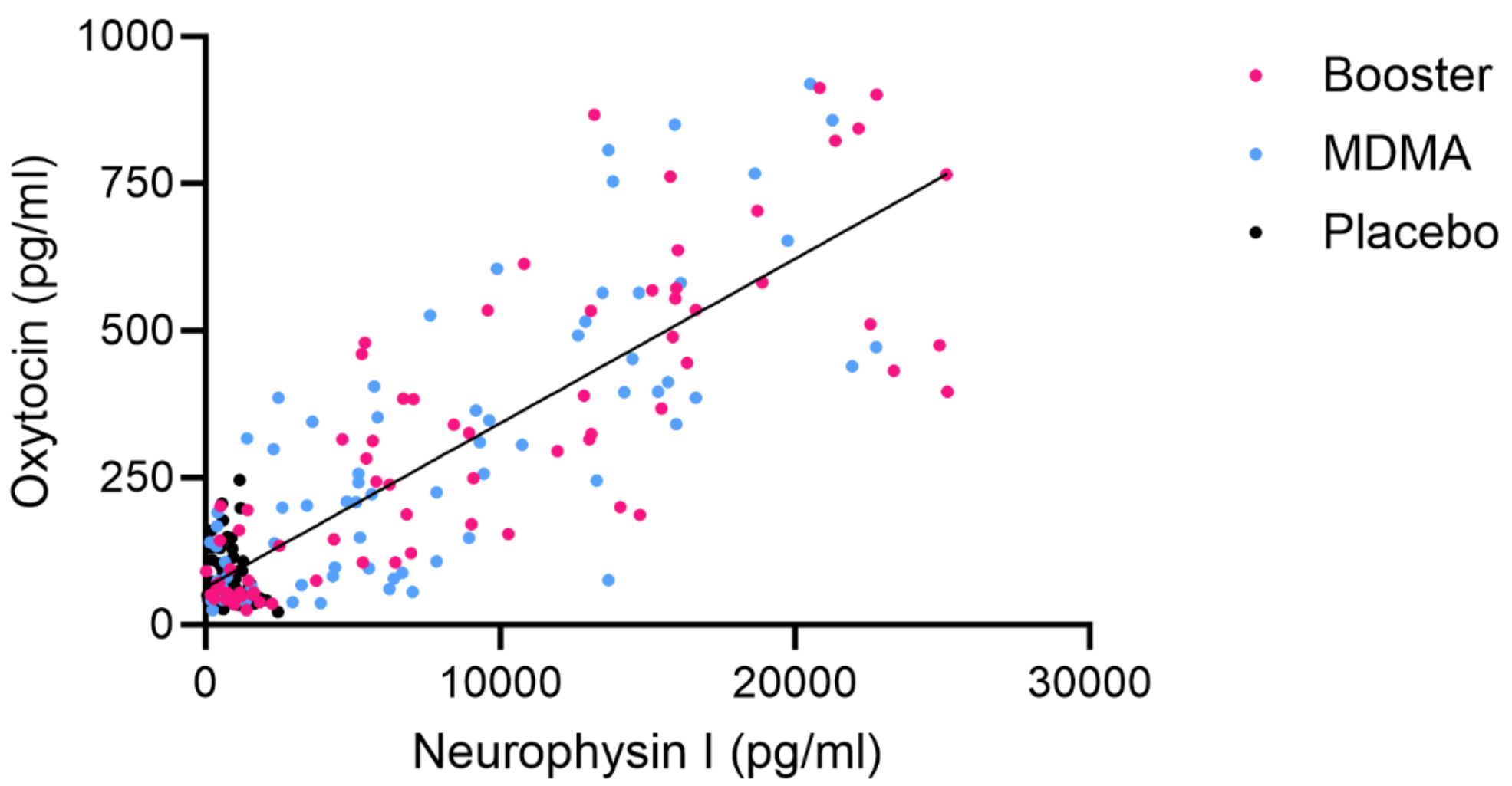

Figure S5 Correlation between oxytocin and neurophysin I plasma levels. Increases in oxytocin levels significantly correlated with increases in neurophysin I. Pearson correlation coefficients and p-values are r = 0.86 and *p<0.0001*. Oxytocin data from the first four participants were excluded from this analysis due to low oxytocin concentrations after MDMA administration, despite increasing neurophysin I concentrations. We expected a preanalytical issue affecting oxytocin specifically because samples from four subsequent participants that were stored and handled together were affected. *n*=19

2.5.2 Table S10: Mean plasma concentrations of oxytocin and neurophysin I over time

Condition	Time point	Booster	MDMA	Placebo	$F_{2, 44}$	*p*	Booster - MDMA	Booster - Placebo	MDMA - Placebo
	h	mean ± SD	mean ± SD	mean ± SD					
**Oxytocin[I] (pg/ml)**									
	0	72 ± 48	93 ± 72	82 ± 51	2.63	0.086	NS	NS	NS
	2	417 ± 259	540 ± 235	77 ± 38	31	<0.001	NS	***	***
	4	500 ± 232	359 ± 169	74 ± 48	44.6	<0.001	**	***	***
	6	290 ± 172	171 ± 114	73 ± 50	33.2	<0.001	***	***	**
**Neurophysin I (pg/ml)**									
	0	834 ± 586	785 ± 785	746 ± 553	0.287	0.752	NS	NS	NS
	2	11329 ± 8793	13359 ± 6283	764 ± 572	36.6	<0.001	NS	***	***
	4	15235 ± 5778	11281 ± 4529	659 ± 394	117	<0.001	***	***	***
	6	9671 ± 3979	5628 ± 2909	687 ± 387	95.5	<0.001	***	***	***

MDMA, 3,4-methylenedioxymethamphetamine; SD, standard deviation. The MDMA condition consisted of a MDMA single dose (120 mg), the Booster condition was a 180 mg split dose (120 mg followed by 60 mg after 2 hours). Repeated Measures Analyses of Variance (ANOVAs), followed by Tukey post hoc tests were used. Results are shown as mean ± SD total scores; *p<0.05; **p<0.01; ***p<0.001; NS, not significant. [I]Oxytocin data from the first four participants were excluded from this analysis due to low oxytocin concentrations after MDMA administration, despite increasing neurophysin I concentrations. We expected a preanalytical issue affecting oxytocin specifically because samples from four subsequent participants that were stored and handled together were affected resulting in n=19. n=23

2.6 Autonomic measures

2.6.1 Table S11: Vital parameters

Condition		Booster	MDMA	Placebo	$F_{2, 44}$	*p*	Booster - MDMA	Booster - Placebo	MDMA - Placebo
Systolic blood pressure, mmHg	E_{max}	154 ± 11	150 ± 14	124 ± 14	127	<0.001	NS	***	***
	Range	94–177	88–177	88–159					
Diastolic blood pressure, mmHg	E_{max}	88 ± 7.1	86 ± 7.2	75 ± 8.7	58	<0.001	NS	***	***
	Range	54–102	48–99	44–95					
Heart rate, bpm	E_{max}	95 ± 18	95 ± 19	78 ± 9.1	21	<0.001	NS	***	***
	Range	49–133	45–157	44–100					
Rate pressure product, bpm x mmHg	E_{max}	13692 ± 2715	13934 ± 3410	9278 ± 1593	46	<0.001	NS	***	***
	Range	5243–19478	5202–24101	4900–12885					
Body temperature, °C	E_{max}	37.6 ± 0.4	37.5 ± 0.3	37.2 ± 0.3	12.8	<0.001	NS	***	**
	Range	36.0–38.2	36.1–38.0	35.6–38.1					

Values are shown as mean ± standard deviation for E_{max} and minimum–maximum for range. MDMA, 3,4-methylenedioxymethamphetamine; E_{max}, maximal effects; mmHg, millimeter mercury; bpm, beats per minute. The MDMA condition consisted of a MDMA single dose (120 mg), the Booster condition was a 180 mg split dose (120 mg followed by 60 mg after 2 hours). Repeated Measures Analyses of Variance (ANOVAs), followed by Tukey post hoc tests were used. **p*< 0.05, ***p*< 0.01, ****p*< 0.001; *n*=23.

2.6.2 Figure S6 Vital parameters over time

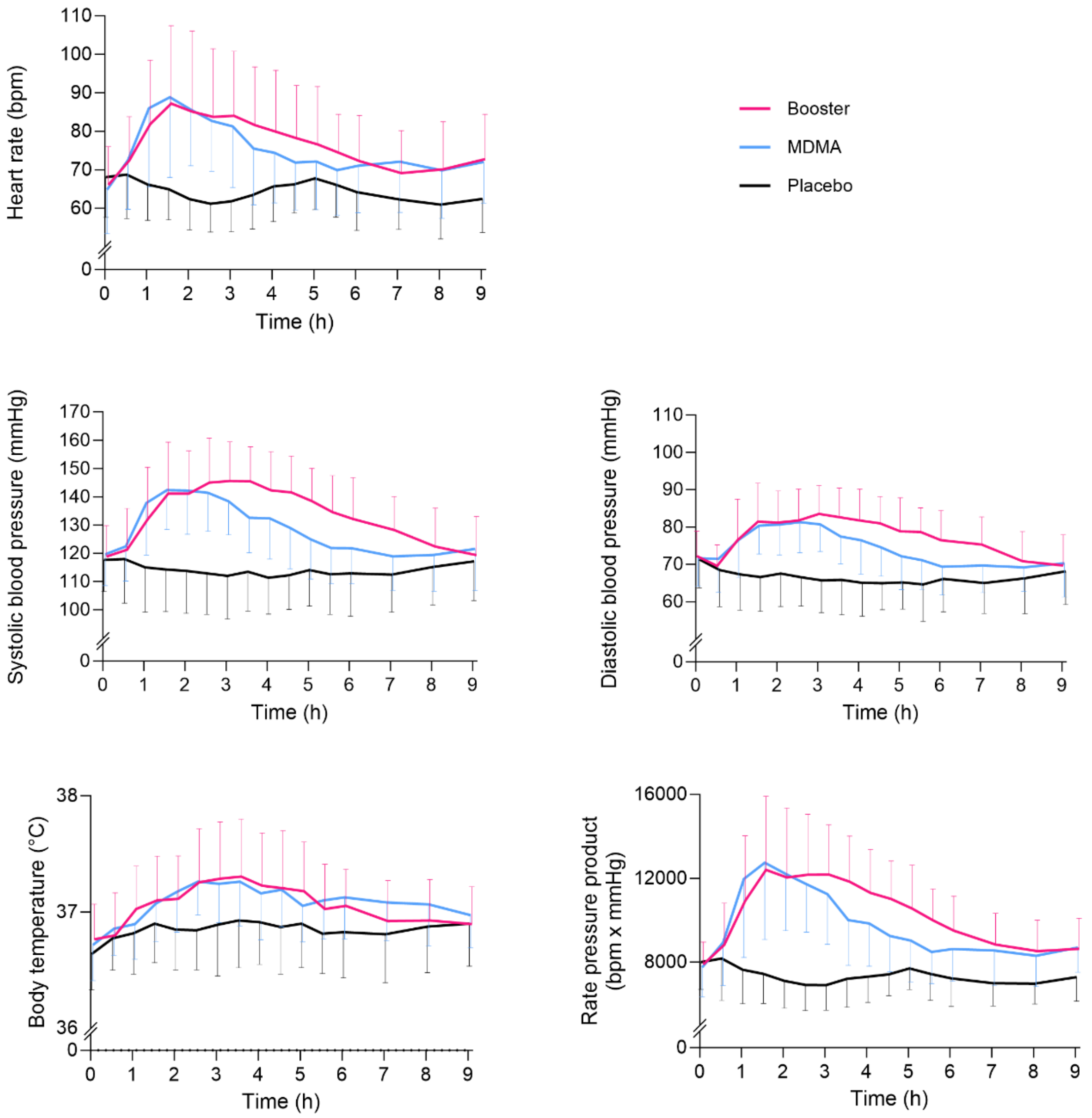

Figure S6 Vital parameters over time for each condition. Data are mean with standard deviation as error bars. *n*=23.

2.6.3 Table S12: Blood chemistry and cell counts before and after the study

	Physiologic range	Screening	End of Study	t	*p*
		mean ± SD (range)	mean ± SD (range)		
**Kidney and liver function**					
Crea, µmol/L	<97	76 ± 16 (53–113)	76 ± 18 (57–122)	–0.0489	NS
GFR, ml/min	>90	102 ± 20 (58–135)	103 ± 19 (53–133)	–0.124	NS
ALT, U/L	<59	22 ± 12 (11–60)	20 ± 7.2 (5.0–32)	0.833	NS
**Blood cell counts**					
WBC, 10^9/L	3.5–10.0	6.2 ± 1.8 (3.5–9.9)	6.2 ± 2.3 (3.2–14.2)	0.176	NS
RBC, 10^{12}/L	4.2–6.3	4.5 ± 0.4 (4.0–5.5)	4.5 ± 0.4 (3.7–5.1)	1.14	NS
Hb, g/L	120–180	141 ± 12 (126–177)	139 ± 13 (114–159)	1.18	NS
Tc, 10^9/L	150–450	249 ± 52 (116–338)	249 ± 53 (136–349)	0.0515	NS

Crea, Creatinine; GFR, Glomerular filtration rate; ALT, Alanine aminotransferase; WBC, White blood cell count; RBC, Red blood cell count; Hb, Hemoglobin; Tc, Thrombocytes; SD, standard deviation; NS, not significant. t- and p-values refer to two-sided paired t-tests. *n*=23.

2.7 Pharmacokinetics

The infinite AUC (AUC_∞) was not calculated and is not reported because the observation period was not long enough to cover >80% of the total AUC leading to large proportions of extrapolation. Also, in several cases, the terminal elimination rate constant could not be determined because concentrations were still high and not yet showing a linear decline at the last observation time point. If the adjusted r^2 for the determination of λ_z was <0.8, the parameters $t_{1/2}$, clearance, and volume of distribution were excluded from the summary statistics. For MDA, $t_{1/2}$ was not determined, because concentrations were still rising in most participants after 9 hours.

MDMA showed a dose-proportional increase in C_{max} and AUC_{0-9}, while this was not the case for HMMA, possibly indicating a saturation in this metabolic pathway.

2.7.1 Figure S7 Mean plasma concentrations of MDMA and its metabolites over time

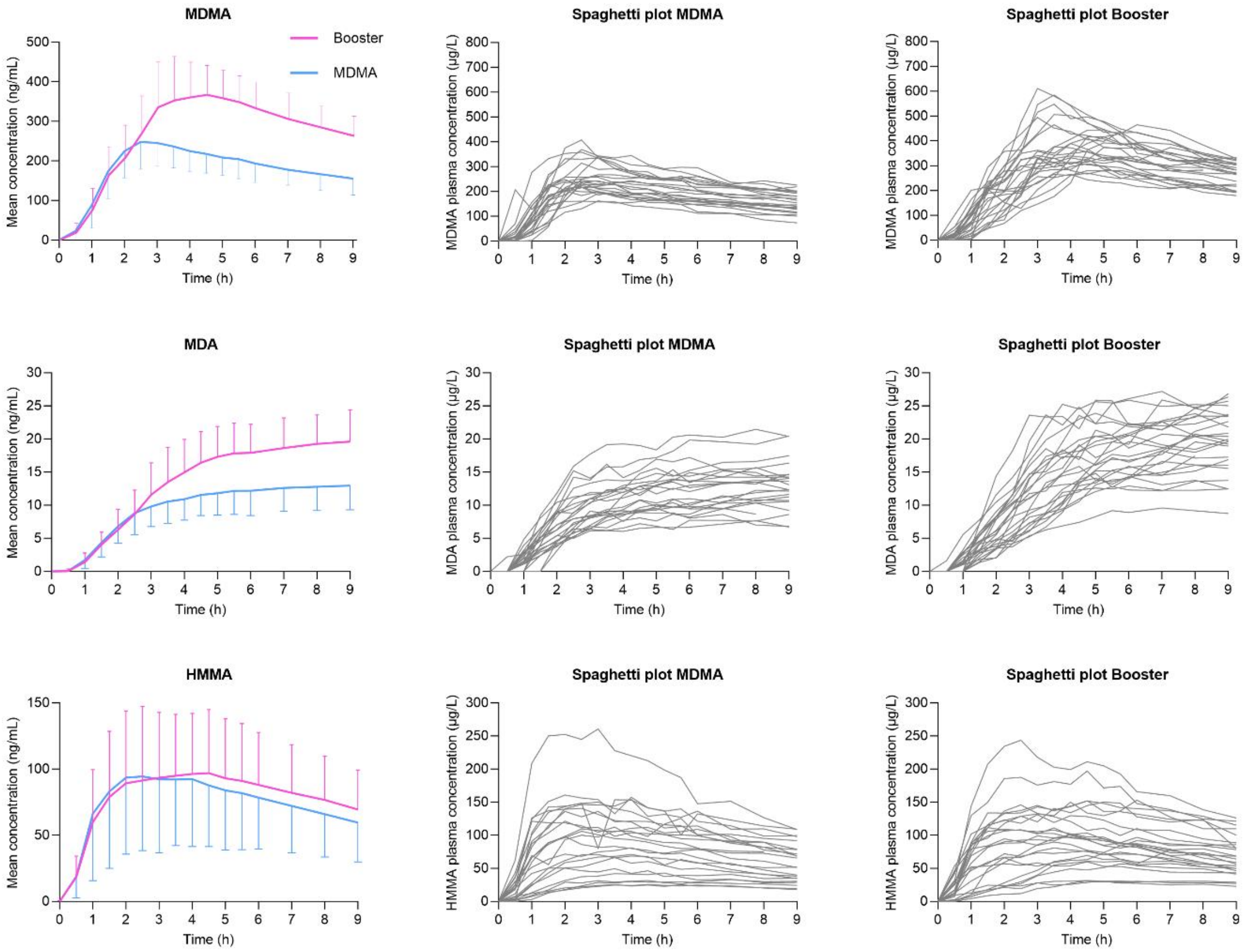

Figure S7 Plasma concentrations of MDMA and its metabolites over time. MDMA, 3,4-methylenedioxymethamphetamine; MDA, 3,4-methylenedioxyamphetamine; HMMA, 4-Hydroxy-3-methoxymethamphetamine. The top row of graphs describes MDMA concentrations, the middle row MDA concentrations and the lowest row HMMA concentrations. The first plot in each row shows mean concentrations with standard deviation as error bars. The second and third plot show individual concentrations for the different conditions. The MDMA condition consisted of a MDMA single dose (120 mg), the Booster condition was a 180 mg split dose (120 mg followed by 60 mg after 2 hours). *n*=25 for the Booster condition, *n*=23 for the MDMA condition.

3. Literature

1 Janke W, Debus G. Die Eigenschaftswörterliste. Hogrefe: Göttingen.; 1978.
2 Zerssen DV. Die Beschwerden-Liste. Münchener Informationssystem. Psychis: München; 1976.
3 Zerssen DV, Petermann F. Beschwerden-Liste: Revidierte Fassung. Hogrefe: Göttingen; 2011.
4 Goldberg DP. The detection of psychiatric illness by questionnaire: A technique for the identification and assessment of non-psychotic psychiatric illness. Oxford U. Press: Oxford, England; 1972.
5 Romppel M, Braehler E, Roth M, Glaesmer H. What is the General Health Questionnaire-12 assessing? Dimensionality and psychometric properties of the General Health Questionnaire-12 in a large scale German population sample. Compr Psychiatry. 2013;54(4):406-13.
6 Schmitz N, Kruse J, Tress W. Psychometric properties of the General Health Questionnaire (GHQ-12) in a German primary care sample. Acta Psychiatr Scand. 1999;100(6):462-8.
7 Tennant R, Hiller L, Fishwick R, Platt S, Joseph S, Weich S, et al. The Warwick-Edinburgh Mental Well-being Scale (WEMWBS): development and UK validation. Health and Quality of Life Outcomes. 2007;5(1):63.
8 Lang G, Bachinger A. Validation of the German Warwick-Edinburgh Mental Well-Being Scale (WEMWBS) in a community-based sample of adults in Austria: a bi-factor modelling approach. Journal of Public Health. 2017;25(2):135-46.
9 Morin CM, Belleville G, Bélanger L, Ivers H. The Insomnia Severity Index: psychometric indicators to detect insomnia cases and evaluate treatment response. Sleep. 2011;34(5):601-8.
10 Dieck A, Morin CM, Backhaus J. A German version of the Insomnia Severity Index. Somnologie. 2018;22(1):27-35.
11 Luethi D, Rudin D, Straumann I, Thomann J, Avedisian I, Liechti ME, et al. Derivatization-free determination of chiral plasma pharmacokinetics of MDMA and its enantiomers. J Chromatogr B Analyt Technol Biomed Life Sci. 2024;1238:124123.
